# What works for peer review and decision-making in research funding: a realist synthesis

**DOI:** 10.1186/s41073-022-00120-2

**Published:** 2022-03-04

**Authors:** Alejandra Recio-Saucedo, Ksenia Crane, Katie Meadmore, Kathryn Fackrell, Hazel Church, Simon Fraser, Amanda Blatch-Jones

**Affiliations:** 1 Wessex Institute, National Institute of Health Research Evaluation, Trials and Studies Coordinating Centre, University of Southampton, Alpha House, Enterprise Road, Southampton, Southampton, SO16 7NS UK; 2 School of Primary Care, Population Sciences and Medical Education, Faculty of Medicine, University of Southampton, Southampton, SO17 1BJ UK

**Keywords:** Peer review, Decision-making in research funding, Grant allocation, Realist synthesis, Research on research, Health research

## Abstract

**Introduction:**

Allocation of research funds relies on peer review to support funding decisions, and these processes can be susceptible to biases and inefficiencies. The aim of this work was to determine which past interventions to peer review and decision-making have worked to improve research funding practices, how they worked, and for whom.

**Methods:**

Realist synthesis of peer-review publications and grey literature reporting interventions in peer review for research funding.

**Results:**

We analysed 96 publications and 36 website sources. Sixty publications enabled us to extract stakeholder-specific context-mechanism-outcomes configurations (CMOCs) for 50 interventions, which formed the basis of our synthesis. Shorter applications, reviewer and applicant training, virtual funding panels, enhanced decision models, institutional submission quotas, applicant training in peer review and grant-writing reduced interrater variability, increased relevance of funded research, reduced time taken to write and review applications, promoted increased investment into innovation, and lowered cost of panels.

**Conclusions:**

Reports of 50 interventions in different areas of peer review provide useful guidance on ways of solving common issues with the peer review process. Evidence of the broader impact of these interventions on the research ecosystem is still needed, and future research should aim to identify processes that consistently work to improve peer review across funders and research contexts.

**Supplementary Information:**

The online version contains supplementary material available at 10.1186/s41073-022-00120-2.

## Background

Peer review is the *de facto* method of determining the best research to tackle today’s societal burdens and has been so since the Second World War [1]. The decision-making processes of research funding allocation supported by peer review are fundamental to the discourse of science as they ensure that funding organisations are held accountable for the significant investments they choose to make into a rapidly evolving and highly competitive research landscape. These processes also help ensure that researchers uphold excellent standards when designing and delivering research and applying for funds [2, 3]. However, research itself has shown that, if left unmonitored or unchanged, this complex social system for allocating funding (for the most part subjectively) to a small portion of ‘only the best’ research ideas out of many can become unreliable, costly and burdensome for the stakeholders involved (the applicants, reviewers, funders and end-users) [4] and lead to waste of research effort [5].

Issues surrounding peer review in research funding have long been reported in the literature [6]. Among these is the administrative workload of the application process [2] and the value of this researcher effort for funders when operating in a climate of high competition and low success rates [7]. The impact of this together with the effects of inflation and application demand on funders’ budgets [8] means that around 75% of the workload involved in peer review falls onto applicants and is unproductive [2, 9]. Peer review may also become problematic to reviewers balancing reviewing activity with their own research and faculty work [10, 11], while for funders the challenge lies in recruiting enough experts to review a growing number of submissions and in the costs of processing submissions and convening panels [2]. Despite this, evidence suggests that aspects of peer review such as the scoring system are in fact poor predictors of research performance [12] and that social phenomena, such as ‘cronyism’, ‘cognitive particularism’ and ‘anti-innovation bias’, are factors that can unintentionally affect the objectivity of decisions in review panels and funding committees [2].

The last two decades have seen a demand for funders to commission more research into improving funding allocation processes and for inefficient practices to be replaced by simpler and better mechanisms of allocating research funds [13]. Still, there is a lack of large-scale experimental studies on the efficacy of these interventions aimed at improving funding processes, with previous randomised trials and meta-analyses focusing on peer review in journal publication [14] – the outcomes of which cannot always be translated to research funding. While the need for trials and prospective studies on interventions remains [2, 15], there is also rationale to provide guidance for stakeholders planning future research or interventions on *what has already been shown to work* in peer review (to enhance processes or solve issues).

## Methods

A systematic map of the literature identifying evidence of efficient and good-quality peer review in health research funding [15] highlighted the need for an in-depth evaluation of innovative approaches to research funding practice, and consideration for the specific research contexts in which these could work successfully. This led to our decision to conduct a realist synthesis [16] identifying past interventions from stakeholders that aimed to enhance the processes of research funding allocation. The synthesis was conducted in six stages, in accordance with the Realist And Meta-narrative Evidence Syntheses: Evolving Standards (RAMESES) publication standards for realist systematic reviews [17].

### Stage 1: scoping and defining programme theory statements

Training on realist synthesis by Justin Jagosh [18] and consultations with Shephard and co-authors of the previously published systematic map of peer review [15] informed the initial scoping strategy for this work (Supplementary file, Search strategy in Medline (OVID)) and development of two high-level programme theory statements (hypotheses; see below) to use for appraisal of the literature (Supplementary file, Programme theory statements).

In realist approaches there are differences in the way in which programme theory statements have been conceptualised [19, 20]. Due to the complexity of this synthesis, we constructed programme theories in the broader sense, providing explanations as to which interventions in the peer review and funding decision-making process can result in reduction of two high-level issues (bias and burden):

A. *If there is peer review (as a process), there is bias and burden to some or all stakeholders.*
B. *If variations (interventions) to the typical peer review process are introduced, then bias and burden can be reduced.*

This approach allowed us to capture relevant literature and, simultaneously, identify other interventions that enhanced or improve other aspects of the funding process. Based on the programme theory statements, if a publication did not provide enough information on the need for change in peer review, or on the intervention and/or its mechanisms for making changes in peer review, it was excluded.

### Stage 2: literature search

Database searches were conducted in December 2019. The search strategy used by Shepherd and colleagues [15] to provide a systematic map of peer review interventions in health research funding was adapted to incorporate: (i) evidence from outside the healthcare sector (e.g., social sciences, art and the humanities, life sciences, hard sciences and engineering, environmental sciences and economics); (ii) qualitative, quantitative, and mixed-methods studies providing empirical evidence on experimental interventions; (iii) studies and reports of outcomes of piloted, recommended or implemented interventions, (iii) editorial and opinion letters, and (iv) other grey literature, including reports, audits, theses, and blogs. The following databases were searched for relevant records: Medline (Ovid); Embase (Ovid); Cochrane Database of Systematic Reviews; Cochrane Central Register of Controlled Trials (CENTRAL); Database of Abstracts of Reviews of Effects; Psychinfo (Ebsco); Web of Science; IEEE/IEEE*Xplore*; ProQuest; arXiv.org; and Delphis (a University of Southampton Library database). The search strategies were tailored to each search engine used (see Supplementary file, Search strategy in Medline (OVID) for examples of search terms). No restrictions were placed on publication dates.

Publications were included if they a) explicitly described any (empirical or implemented) interventions relevant to peer review and decision-making in research funding allocation, b) addressed one or both programme theory statements, c) were available in full text and d) were published in English. Records were excluded if a) they only offered background and already widely publicised information (e.g., describing the well-known strengths and limitations of peer review) without specifying an intervention, b) interventions were already part of standard practice, or c) full texts of abstracts were not found. Publications relevant to journal peer review were also excluded, except for one [21] which reported a randomised trial on the efficacy of reviewer training as an intervention that we deemed to be conceptually transferrable to peer review in research funding. Following completion of the database search, the search strategy was extended in April 2020 to include manual web searches of the current peer review and funding practices employed by various international funders (full list provided in Supplementary file, Update on recent funder interventions). The purpose of this search was to obtain the most up-to-date information (up to 2020) on any long-term outcomes from the interventions extracted from the original publication sample (i.e., current evidence of funder implementations or reform).

### Stage 3: appraisal of the evidence

Records identified through database searches were exported into EndNote X9.2 (Clarivate, UK) for record storage, duplicate removal, full text retrieval, and eligibility screening of references identified from electronic databases and manual searches. Five authors (ARS, KC, ABJ, KM and KF) appraised the evidence by screening the publications, testing the relevance of the records against the programme theory statements, determining whether study methodologies were appropriate for the study designs, and confirming the validity of the sources (i.e., peer-reviewed journals, scientific magazines, or blogs from recognised organisations and institutions). Interrater agreement was tested on 250 random publications and was 90%. Disagreements were resolved through discussion and consensus.

### Stage 4: data extraction

Data extraction from all included publications was conducted mainly by AR and KC, with the rest of the authors contributing to the extraction of 10% of the literature. Data extracted into a structured Excel spreadsheet was summarised for each individual study (see Supplementary file: Summary of findings of publications reviewed). Information supporting the appraisal of the data was also extracted and summarised (Supplementary file: Summary of findings of publications reviewed), then used to inform stage 5, where the Contexts, Mechanisms and Outcomes (CMOs) of individual publications were aggregated for ease of analysis and discoverability. Systematic reviews identified from the database search that captured interventions relevant to the programme theory statements, and reported multiple outcomes for stakeholders, were included but not used to extract individual CMO links.

### Stage 5: analysis and synthesis

Analysis and synthesis were carried out by AR and KC, with consultation from the rest of the authors, to ensure robustness of interpreted data and clarity of arguments. Publications were characterised according to type, research design (where applicable), content, stakeholder involvement/engagement, research field and location. The proportion of evidence for each category is summarised in Supplementary file, Characterisation of publications. The different types of interventions captured in the publication summaries were listed and coded (e.g., interventions related to applicant performance and publication metrics were coded under ‘bibliometrics’), and their frequencies sorted in descending order according to frequency of citation in the literature (Supplementary file, Types of interventions by frequency of use). CMO Configuration (CMOC) informed the main synthesis, which was structured to answer the core realist question of *what works, for whom, and how?* (Table 1). This involved integrating a high number of specific research scenarios with different types of stakeholders/interventions and identifying the outcomes of the interventions. The following definitions were applied to CMOs for this analysis:

- **Contexts**: Areas of peer review and decision-making where the need for change was commonly cited in the literature (i.e., *What were the drivers for change?*).
- **Mechanisms**: The high-level changes made to the peer review process (*What areas needed addressing to solve the issues?*) via specific stakeholder-interventions from one or more stakeholder
- **Outcomes:** The consequences of the stakeholder-specific interventions, including long- and short-term outcomes (*What happened as a result and what were stakeholders' reactions?*) and the stakeholders (*For whom was there overall benefit?*). In this review, short-term outcomes were defined as research outputs or funding outcomes of individual pilot schemes/interventions and long-term outcomes were defined as recommendations for or changes to funding practice and/or the wider research ecosystem. Unintended consequences of interventions, where captured, were also included (*Did the changes create burden/benefit elsewhere in the peer review process?*).

**Table 1:** Contexts-Mechanisms-Outcomes Configurations of past stakeholder interventions in peer review and decision-making for research funding

Contexts	Mechanisms		Outcomes			
Common issues in peer review and decision-making	High-level changes made to the peer review process by stakeholders	Specific interventions implemented by stakeholders (*n* = 50)	Intervention outcomes (long- and short-term)	Stakeholders involved in/affected by interventions	Unintended consequences	Publications
*What were the drivers for change?*	*What particular area needed addressing to solve the issues?*		*What happened as a result and what were stakeholder reactions?*	*For whom?*	*Did the changes create burden/benefit elsewhere in the peer review process?*	
**1. Scientific, economic and social benefit of research**	Promoting collaboration between academic research and public sponsors of research	A government-led ‘audition system’ for matching individual research groups to relevant sponsors of social priorities and industries	Increased emphasis on the social relevance and impact of funded research across academia and industry	General public Research sectors Funders		[29–30]
	Enhancing use of metrics to assess research impact	Incorporation of ‘altmetrics’ into decision-making and facilitating international collaboration to achieve open access infrastructure to researcher metrics	Repositories (e.g., Lattes database, Researchfish) serve as examples of open tracking of research performance and impact on a national level to inform funding decisions			
**2. Career stability of researchers**	Considering how changes to funding policy impact the stability and progression of researcher careers	University College Union’s open letter to Research Councils UK recommending interventions that focus on benefiting researchers and subsequent nation-wide campaigns to abolish casual contracts and promote contract continuity	University College Union campaign documented resulting changes to contract culture (e.g., more permanent contracts) and career stability has gained momentum in wider research community conversations	Higher and further education sectors in the UK		32–33
**3. Funding and support for innovative research**	Minimising emphasis on researcher track record and promoting reviewer autonomy in decision-making	Masking applicants’ institutional affiliations/track record from reviewers and allocating them a ‘Golden’ ticket to fund one proposal of their choice	Anonymity of applications allowed reviewers to focus on research ideas that would have otherwise not been funded and encouraged early-career researchers to propose innovative ideas	Early-career researchers Funders Reviewers		[34–39]
	Creating dedicated funding streams	Funding of high-risk, high-reward research from early-career researchers	New Innovator and Early Independence awards at the National Institutes of Health			
	New approaches to ‘balance’ funding decisions	Use of the Delphi method to promote innovation in niche areas of research	Removing ‘group think’ from decision-making encouraged more funding of innovative ideas and assembling the Delphi panel of experts saved administrative time			
**4. Selection and accountability of reviewers**	Creating reviewer registries and existing diversifying reviewer pools	Use bibliometric data to assess existing/create new registries of multidisciplinary and scientifically active reviewers	Bibliometric data helped reveal gaps in expertise needing recruitment of more experts who remain active in research	General public Funders Academic researchers Reviewers		[41–45]
	Using bibliometrics to automate the reviewer selection process	A semi-automatic tool for selecting experts based on their Pubmed IDs	More targeted selection of review candidates and rejection of unsuitable reviewers		Using Pubmed IDs as proof of reviewer expertise may encourage ‘performance bias’	
	Enhancing decision-making processes informed by peer review	Periodic audit of research charities to ensure their funding practices align to ‘core principles’ of peer review	Stronger conflict of interest policies, independent review monitoring, reviewer rotation and transparency of review practices and reviewer identities			
**5. Allocation of reviewers to proposals**	Enhancing reviewer-proposal matching based on expertise and funder guidelines	Semi-automatic tool for matching reviewers to proposals based on bibliometric data	Effective matching of reviewers to all proposals and improved accessibility for reviewers and programme officers	Funders Reviewers		[42, 46–50]
		Network flow theory algorithm using funder guidelines for reviewer selection (e.g., number needed per proposal)	Successful and balanced matching of reviewers to proposals without conflict of interest			
	Increasing the number of reviewers assigned to proposals	Assign more than two reviewers to each proposal and more than one proposal for each reviewer	Increased interrater reliability		Assinging more proposals to each reviewer can lead to ‘reviewer fatigue’ and assigning more than two reviewers to each proposal may not be realistic for smaller funders	
	Involving applicants in the review process	Applicants review each others’ proposals with the incentive of achieving higher scores for honesty and reliability	High quality and reliability of review and a ‘highly motivated’ reviewer pool that required less administrative effort from programme officers		Requiring group consensus to achieve interrater reliability may discourage applicants from proposing innovative ideas.	
**6. Quality and reliability of peer review**	Training reviewers to improve interrater reliability	Self-taught training material on peer review in publication or research funding (explaining review criteria and the scoring system) for novice and experienced reviewers	Overall significant improvement in review reliability in terms of identification of errors/recommending manuscripts for rejection (for publication review) and understanding of the scoring system and time spent studying review criteria (for grant review)	Funders Reviewers Applicants		[22, 53–55, 96]
	Employing two independent review panels to assess proposals	Using interpanel agreement and the impact of decisions to determine the reliability of review	Increased interrater agreement of funding decisions.		Reduced emphasis of reviewers on the track record	
	Simplifying scoring systems	Use dichotomous (yes/no, 0/1) scores rather than ‘scale-based’ (‘not at all’/‘somewhat’/‘definitely’, 0/1/2) review scores	An equally reliable but simpler scoring system of reviewing full proposals			
**7. Patient and public involvement in funding decisions**	Promoting community engagement of research through applicant and reviewer training	Collaboration of public members, academic experts, patient representatives and reviewers to analyse barriers to funding more community-based research	A community engagement framework at the National Institutes of HeALTH (definition of ‘community engagement’, strategies for researcher training, and guidance for reviewers)	Funders Applicants End-users (the community)		[57–60]
	Involving public members, patients and health advocates in decision-making	Re-reviewing expert-reviewed proposals by community representatives trained in peer review for relevance of proposals to community needs (i.e., two-tier review system)	Funding of proposals that meet both scientific and community criteria and success of resulting research in the form of external grants and peer-reviewed publications			
		Two-tier review system involving expert-led review and review by a ‘wider stakeholder’ group (patients, public members)	Increased translational and social relevance of funded research and inclusion of wider stakeholders in further funding calls			
		Two-tier review system involving review of scientific merit and community engagement of research based on criteria of ‘involvement, priority, and benefit’	Increased emphasis of funding decisions on scientific merit and community engagement criteria, instead of research budget			
		Two-tier review system led by experts and research ‘consumers’ (survivors or patient advocates), who were also involved in decision-making.	Overall, the intervention received positive feedback from all stakeholders		Involving consumers in peer review and decision-making should involve ensuring that the scientific merit of funded research is not compromised	
**8. Unnecessary burden on applicants**	Shortening applications and limiting technical detail	Reduce the word limit of applications and focus them on the research question, methodology, budget, healthcare partnerships and potential impact of research	A 1200-word proposal took applicants on average only seven days to prepare, and more applications were shortlisted/received invitations to interview	Applicants Early-career researchers Faculty staff Reviewers		[34, 63–74]
		Shortening the research plan of large project proposals from 25 to 12 pages.	A shorter R01 application format at the National Institutes of Health			
		Two-page synopsis of the ‘central research idea’	Implementation of the synopsis format in subsequent funding calls at the National Science Foundation, followed by a request from reviewrs for a four-page format			
		Two-page summary of anonymised proposals	Increased funding of innovative proposals at the Villum Foundation (CMOC 3)			
	Improving feedback for applicants	Using decision software to generate a feedback summary	ProGrid decision software provided ‘meaningful feedback’, which was well received by applicants			
		Providing applicants with multiple rounds of online peer feedback	Proposal quality is significantly improved when feedback is given early in the application process			
		Providing applicants with feedback after application triage, before peer review	RAND Europe recommendation following a consultation exercise			
	Streamlined funding process	Shortening the review window, convening funding committees earlier and extending resubmission deadlines	A streamlined funding cycle was implemented across the NIH as it gave applicants more time to address panel feedback and submit resubmissions without having to wait for the next cycle			
	Open access to peer review	Make reviewers’ comments on proposals accessible to other reviewers	Cross-community critique of reviewer comments gives them the opportunity to modify them, promoting transparency and accountability of peer review			
	Improving applicants’ grant writing skills	Funder outreach in the form of talks and grant writing workshops at universities	Helping researchers write better proposals that align with the funder’s mission achieved high success rates			
		Publicising outcomes of funding cycles, discussion of submission policies, mentoring and networking, explaining the funding process, and helping write stronger proposals	The National Science Foundation made a long-term plan to increase outreach activities across HEIs		Publicising institutional submission/success rates helps create a culture of open research	
	Educating applicants on the funding process	Internal student-run funding programme	Educating PhD students and allowing them to engage in peer review gave them valuable experience of how research funding works			
		Internal programme to improve the quality of education provided by faculty staff and reduce for them the burden of grant writing	The programme led to internal and external funding of research, research publication and dissemination, and an increase in external investment into education			
		*An Educational Research Methods* course	Educating applicants in research methods contributed towards improving the quality of proposals			
	Promoting funding of new investigators	Funding scheme for new investigators	The ‘New Investigator Award’ at the National Institutes of Health equalised success rates for new and established investigators in the pilot round, leading to its implementation and addition of the ‘Early Stage Investigator Award’ and ‘Pathway to Independence Award’.			
**9. Unnecessary burden for reviewers**	Optimising review structures	Larger study sections at the National Institutes of Health (e.g., covering both clinical and basic research), new monitoring systems, and shorter proposals	Less pressure on review panels	Reviewers Funders		[44, 64, 67, 68, 75, 76, 81]
	Virtual panels and rotating reviewers	Replacing in-person review with virtual panels, breaks from study sections, asking long-serving reviewers to temporarily step down, and employing ‘ad hoc’ reviewers	Reduced administrative cost to funders, reduced reviewer fatigue, reliable peer review, fresh insight from ad hoc reviewers into review process		Costs of investment to conduct virtual funding panels. Difficulties in adopting the practice because of high reviewer rejection rates.	
		Funder investment into virtual technology to standardise the practice of remote review and adding more reviewers to panels	The National Science Foundation published these plans as part of their mission to reduce reviewer fatigue. The practice also reduced funder costs without consequence to interrater reliability or quality of discussions at Marie Sklodowska-Curie Actions and the American Institute of Biological Sciences			
	Application triage	Incorporate, where possible, application triage into peer review to manage application demand against available funding	Recommendations from RAND to enhance peer review by filtering out low-chance applications in triage and improving feedback for applicants was aimed at all research funders			
**10. Unnecessary burden for funders**	Controlling application demand	Limits re-submissions per applicant per cycle and for the weakest proposals	Limiting R01 re-submissions was part of the long-term reorganisation of review structures at the National Institutes of Health	Funders		[63, 65, 68, 77–83]
		Place quotas on new (preliminary) proposals, make full proposals invitation-only, limit invitations from each institution and encourage internal peer review	Submission quotas were implemented long-term by the National Science Foundation and further tightened in response to rising demand		Submission quotas creating the need for internal review at Higher Education Institutions was seen as ‘shifting of burden’ from funder to researcher that would increase workload for and competition among applicants	
		Limit submissions using a cooling-off period between rounds	This approach may be more effective than a ‘multiple proposals strategy’ in increasing success rates			
	Introducing internal peer review	Require applications to be internally reviewed and scored prior to submission to funders	An increase in publication output			
	Virtual technology and automation	Standardise the practice of virtual review, invest in virtual technology and automate application processing	Reduced cost of review and more administrative capacity for reviewer management		Additional cost to the funder of investment into virtual technology	
	Enhancing the reviewer pool	Increasing the number of reviewers per panel, allocating more reviewers per proposal and using group consensus to score applications	Potential reduction in funder burden if demand is also reduced (e.g., with submission quotas)			
	Decision models	Use software (e.g., ProGrid, Teamworker) to evaluate applications based on a ‘performance matrix’ of researcher/proposal variables	Enhanced funding decisions, simplified discussions, and shorter meetings (ProGrid); fairer proposal discussions (Teamworker)			
		Correlate review scores with applicant CV data to predict likelihood of research success	Identification of promising candidates based on research productivity and factors that are unrelated but may create bias (e.g., age, gender)			
	Streamlining the funding process	Shorter applications and simplified scoring	Reduced financial and administrative burden for the funder, and a faster process for applicants		Reduced emphasis on the applicant’s track record	

### Stage 6. Dissemination and consultation

This work has been disseminated at international conferences and research seminars [22–24] and with staff at the National Institute for Health Research Evaluation, Trials and Studies Coordinating Centre in support of the ‘*Busting Bureaucracy: empowering frontline staff by reducing excess bureaucracy in the health and care system in England’* initiative led by the UK government to reduce bureaucratic burden in research, innovation and higher education [25, 26]..

## Results

### Search results

**Fig. 1:**
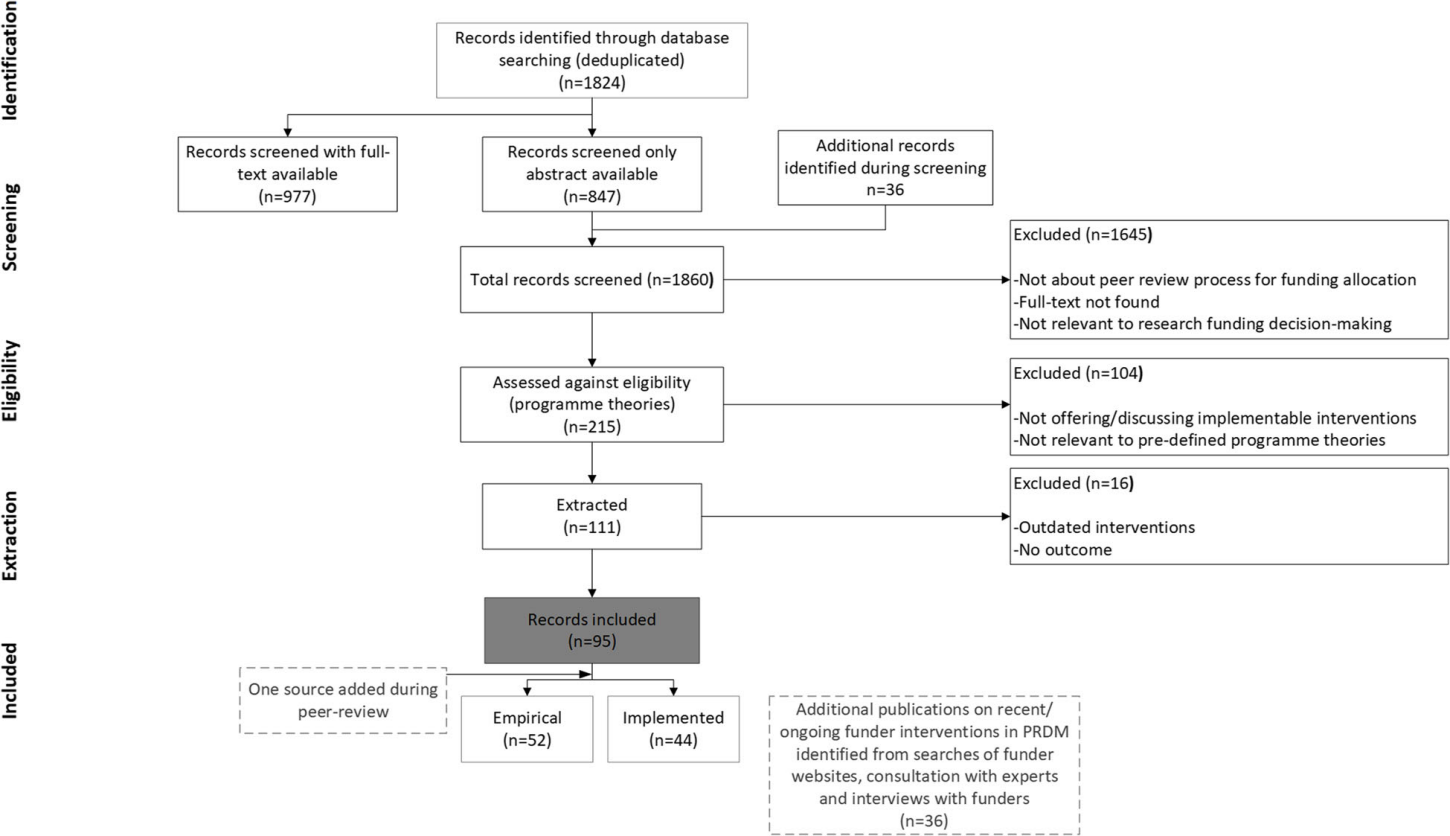
Prisma flowchart: Search strategy to identify publications on interventions in peer review and funding allocation

The publication search strategy returned 1860 records. Of these, 1645 publications were excluded based on the primary criteria (see Methods section). Screening of the remaining 215 articles by ARS and KC resulted in a further 104 publications being excluded for not addressing the programme theory statements, leaving 111 articles. A final screen during extraction resulted in exclusion of 16 articles, leaving a total of 95 publications to include in the synthesis; however, after peer review, one publication that was originally excluded was re-added for providing a relevant CMO link. Figure 1 depicts the results of the original search strategy that included the original 95 publications, with a separate box indicating the additional publication included during peer review.

The manual search of funder websites (see Methods section) identified 36 relevant web sources providing updated information on changes to peer review practice and research commissioned between 2017 and 2020 (Supplementary file, Update on recent funder interventions). Ad hoc consultations with two European funding organisations (The Lundbeck Foundation and the Health Foundation QExchange) provided insight into interventions recently implemented by these funders.

Of the 95 database records (plus one re-added post-review), 51 described empirical interventions and 44 described piloted/implemented interventions (Fig. 1). A total of 60 publications provided CMO links for interventions and 62 outcomes were found in total; of these, 33 were associated with ‘short-term’ intervention outcomes and 29 with ‘long-term’ intervention outcomes (with one publication (RCUK, 2007) reporting more than one outcome).

### Data extraction, main findings, and synthesis

A summary of key information from the 96 publications is available in the Supplementary file (Summary of findings of publications reviewed, Characterisation of publications). Approximately half (52%) of the publications were original research papers – mostly observational studies (17%), pilots (defined as small-scale interventions on specific research calls or funding processes that involve multiple designs/methods including qualitative or quantitative evaluation studies using interviews/focus groups, surveys, or analysis of quantitative data) (15%) and computational models/simulations (14%). Forty-eight per cent were non-research articles, of which 18% offered 'hypothetical' interventions (e.g., opinion pieces or commentaries about interventions yet to be researched) and were not included in CMO analysis as no outcomes could be extracted from them.

Almost half (48%) of the publications were related to health research, 37% to biomedical research and 42% were not specified to a particular research area. Multiple publications were relevant to more than one research field, with the most common overlap between health and biomedical research. Characterisation of publications by ‘stakeholder involvement’ (i.e., identifying the stakeholders that were either authors or the target audience) revealed that 83% of publications featured the funder as the major stakeholder and 51% featured Higher Education Institution (HEIs) researchers, also signifying overlap (both were clearly intensively and collaboratively involved in developing peer review research and practice). Geographically, there was a concentration of articles originating from the USA (54%) and less so from Europe (22%), among other locations (Supplementary file, Characterisation of publications).

A wide variety of interventions was described in the literature (see Supplementary file: Types of interventions by frequency of use), of which the most common interventions related to reviewer selection, rotation, or proposal matching (19%); review criteria (17%); funding allocation (16%); reviewer or applicant training (16%); modelling decision-making and use of the Delphi method (15%).

### Contexts-mechanisms-outcomes configuration

Contexts, mechanisms, and outcomes configurations (CMOC) present the interaction between drivers for change in peer review and decision-making, interventions, and what happened because of those interventions. CMOC analysis provided evidence of intervention outcomes extracted into 10 CMOCs linked to 50 stakeholder-specific interventions to improve various mechanisms of the funding process (Table 1 Context-Mechanism-Outcome configuration). Publications reporting interventions aimed at decreasing unnecessary burden for applicants (CMOC 8), reviewers (CMOC 9) and funders (CMOC 10), triggered 14 stakeholder interventions as captured in the literature. In contrast, only two interventions were found to focus on the ‘Scientific, economic and social benefit of research’ (CMOC 1) and only one on the ‘Career stability of researchers’ (CMOC 2). The common issues reflected in other CMOCs (Funding and support for innovative research, Quality and reliability of peer review, Selection and accountability of reviewers, Allocation of reviewers to proposals, and Patient and public involvement in funding decisions) triggered between 4 and 6 stakeholder interventions. Although CMOCs 1 (Scientific, economic, and social benefit of research) and 2 (Career stability of researchers) are not directly relevant to peer review, they still capture interventions to funding practice (e.g., matching research ideas to sponsors and prolonging research contracts) and as such are relevant to all stakeholders involved in research funding based on peer review processes. Other relevant interventions, for instance, included the creation of research repositories for reviewers to assess researchers’ performances and campaigns to support ECRs.

### Interventions that worked

Intervention outcomes were categorised into short-term and long-term.

#### Short-term outcomes

A third of interventions reporting short-term outcomes aimed at improving reviewer identification, selection, and matching of proposals to reviewers; reviewer training; and increasing the reliability of reviews. Creation of reviewers’ registries based on bibliometric data [27]; automatically assigning reviews based on bibliometric data [28, 29]; creating funding decision support models with bibliometric data [30]; conducting analysis of CV attributes on research productivity of applicants to calculate a probability of receiving funds compared to peer reviewers' scoring assessments [31]; training reviewers [21 and 33]; and having applicants conduct peer review of proposals competing for the same funds [32] have shown promising results in improving funding processes relevant to the organisation where the interventions took place, but all recommendations are tagged with caveats.

The use of bibliometric data to create reviewers’ registries was not viable due to differences in classification systems for research outputs and the non-uniform distribution of experts in different fields. However, bibliometric data used to create a web tool that semi-automatised reviewer selection for fellowship applications improved selection by saving administrator time [28]. Models based on bibliometric data to support reviewers are offered as adjunct and not a replacement of funding decisions based on peer review. Using CV data to calculate probability of success of grant applications and rank applicants was reliable, however it was found to be more appropriate for fellowships due to the emphasis on the individual and level of CV information available, and that it required high level alignment between institutions to generate CV data. Interventions to train reviewers on assessment criteria have shown potential in reducing bias and increasing interrater reliability [33]. Statistical analysis of grant application scores to explore the most appropriate number of reviewers [34] found that applications in fields that are not controversial may not require a high number of reviewers (i.e., if the expectation is that reviewers will on average agree) and that four reviewers is a minimum appropriate number; when the application is more likely to divide reviewers, a larger number of reviewers are required (however recognising that implementing flexible systems that allow selecting different number of reviewers to assess individual applications would require complex proposal management processes that could be tailored for individual calls). Looking at improving the reliability of peer review, achieving greater consensus, and selection fairness through analysis of funding panels and application data have resulted in recommendations for the use of multidisciplinary panels (46; 55), not using researcher-nominated reviewers, increasing reviews per proposal, and having a smaller number of more highly selected reviewers to conduct the majority of reviews within subdisciplines. However, a limitation of the analysis on which these recommendations were based was a lack of outcome measures against which to validate the peer review process.

Interventions to simplify peer review criteria formed another group in the short-term outcomes category [35–39]. Outcomes of interventions that included changing criteria and a simplified process to conduct peer review assessments were observed in the agreement of decisions on 72 applications between panels using an organisation standard system (12 reviewers who met face to face and reviewed 100-page applications) vs simplified systems (a. 7 reviewers who met face to face and reviewed a 9-page shortened application b. 2 reviewers who only reviewed shortened applications independently). Although agreement between decisions made using standard and simplified systems, was below an acceptable threshold (75%), the potential savings from peer reviewer time and travel cost was recognised [38]. Overall, simplifying review criteria has shown potential but the challenge to determine the benchmark against which measures of success are observed remain. Finally, an intervention where applicants became part of the peer review process by assessing proposals from applicants competing for the same funds resulted in a highly motivated pool of reviewers who produced reviews comparable to those conducted in the traditional process. Conflict of interest was controlled by comparing scores and observing whether individual scores agreed with the group. Administrative benefit was observed in reduced time required to find reviewers, but concerns were raised on group score consensus which some believed could discourage innovative ideas [32].

#### Long-term outcomes

Extracted interventions under long-term outcomes covered different components of the funding process. Virtual panel meetings have shown to offer a good alternative to conduct peer review funding decisions. An examination of the effects of panel discussions on scoring, magnitude of score shifts and application discussion time in face-to-face and teleconference funding panel meetings has shown little difference between settings [40]. Retrospective analysis of peer review data investigating whether the review setting has an effect on the review process and outcome measures (overall scientific merit scores, score distribution, standard deviations and reviewer inter-rater reliability) showed that virtual meetings did not significantly decrease discussion time, average overall scores were comparable between the two meeting modes and peer review retained a high level of reliability (note that each application had 10 reviewers and the reliability of ratings increases with an increase in the number of reviewers) [41]. Finally, a survey of reviewers found that the review quality remained in virtual meetings. Furthermore, travel costs associated with face-to-face meetings reduced annual costs of running funding panels. A caveat of virtual panels was the need for investment in technology to support the meetings. Introducing submission quotas has decreased the workload involved in processing applications but has generated an unintended consequence of additional workload to peer review applications within university departments. The potential negative effect can be offset if peer review within HEIs is used to foster collaborations and multidisciplinary research [42]. Another promising type of intervention was that of shortening applications which decreased the workload of applicants, reviewers, and funders by shortening writing time, expediting reviewing, and shortening funding decision time [43, 44]. A combination of shortened and anonymised proposals was perceived to reduce applicant selection bias when relatively unknown researchers received high scores in their applications [45]. Finally, supporting peer review with the Delphi method, randomisation, or golden tickets (where reviewers are able to champion proposals) has shown reduction in selection bias by reducing group think and support funding review efficiency when access to experts in a specialised field is required [46]. Evidence extracted from recent funder website searches also points to incremental changes to peer review processes used in combination with innovative funding processes. The Bill & Melinda Gates Foundation uses blind review to allocate awards for its Grand Challenges Explorations programme where the name and affiliation of applicants are omitted [47], the HRCNZ uses a modified lottery for their ‘Explorer’ grants programme to prioritise proposals [48], and the EPSRC uses ‘sandpits’ in their Digital technologies for health and care programme [49].

### CMOC 1 – research delivers scientific, economic, and social benefit

Two interventions focused on ensuring that funded research delivers societal impact, is tailored to public priorities, and is delivered by teams that have been carefully and transparently selected by funding committees.

#### Increasing collaboration between public sponsors and researchers

A retrospective analysis [50] of an ‘audition system’ adopted by the Japanese government for research grant review enacted changes in law and policy to shift the priorities of academic research from discovery towards social and economic impact. This audition system was structured around a ‘producer’ whose job was to connect researchers with various public and industry stakeholders wishing to make an investment into research targeted at social needs. The premise was that encouraging collaboration between HEIs, and other research sectors already invested in tackling social and economic research needs would help achieve harmony between the sectors and align academic research priorities with those of the public and government, leading to better reflections of public priorities in funding decisions. Anecdotally, there is suggestion of a long-term outcome from this government scheme on the Japanese academic landscape; universities in Japan have since put more focus on collaboration in funding programmes and, as an example, the Core Research for Evolutional Science and Technology programme (CREST) now prioritises funding of innovative research ideas that promise social and economic benefit and a strong team-oriented work ethos. However, the evidence is unclear on whether the audition system as an intervention achieved long-term uptake by HEIs and other research sectors in Japan or elsewhere [50]; what can be gleaned is that the intervention successfully promoted the long-term goal of increasing the social impact of academic research in Japan.

#### Improving metrics to assess potential in funding decisions

According to the National Science Foundation (NSF) [51], use of well-established metrics of research performance and impact (such as the citation index and journal impact factor) to make funding decisions may lead to more robust and reliable funding outcomes if combined with alternative types of metric data (e.g., social media outputs) – the reason for this being that including ‘altmetrics’ should provide funders with more accurate and rounded reflections of scientific activity and impact compared to standard indicators alone [51]. As such, the NSF called for a concerted international effort to facilitate broader funder and researcher access to metric data, so that any calculations used to make funding decisions can be checked (e.g., for bias), reproduced or repurposed by anyone in the research community [51]. While creating one international infrastructure for metrics sharing and analysis is an ideal scenario to achieve this, it would require significant collaboration from funding agencies to align their mechanisms of tracking researcher performance to achieve equality. Some examples of this infrastructure on a national level already exist. The Lattes Database [52] in Brazil, for instance, openly provides metric data on ~ 1.6 million researchers across 4000 institutions. In the UK, the equivalent platform, Researchfish [53], provides funders and the public with highly structured, rich datasets and lay output on the outcomes and impacts of grants awarded by currently 18 national funders (including the NIHR) and many charities.

### CMOC 2 – career stability of researchers

In 2006, Research Councils UK (now the UK Research and Innovation Council; UKRI) published a report titled the ‘RCUK Efficiency and Effectiveness Peer Review project’ [54]. This highlighted their work on four generalisable interventions that the RCUK modelled using their own data, namely: 1) increasing the duration of funded grants (e.g., from 3 to 5 years); 2) introducing HEI application quotas to reduce application demand for research councils; 3) limiting resubmissions; and 4) introducing an ‘outline bid’ (i.e., triage) stage into the submission process to reduce the burden of reviewing full proposals for reviewers. These recommended interventions were met with some constructive criticism from the the University College Union (UCU), who conducted a community-wide survey and published a rebuttal for RCUK with their own recommendations for the funder to focus their interventions on better supporting researchers [55]. These recommendations included: preventing concentration of UK funding to the top 10% HEIs; reducing RCUK funding costs by creating permanent or open-ended contracts for researchers; repurposing small grants for research disciplines that are low-budget but early career researcher (ECR)-rich (e.g., the Arts and Humanities); adopting a meritocratic approach of basing funding decisions on the quality of proposals and not the institutional affiliations of applicants; and promoting funding of multidisciplinary research by increasing reviewer pools and introducing more experts from diverse backgrounds or emerging fields. Since this survey, there is indirect but compelling evidence that it fostered increased interest in career stability within the research community and that positive long-term changes have been enacted within UK academia [56] and as a result of UCU’s ongoing campaign to promote stability and continuity of employment in all education sectors [57].

### CMOC 3 – funding and support for innovative research

Effort to address reported conservatism and ‘anti-innovation’ bias in research funding [58] was reflected in 8% of the publications. The interventions involved focused on empowering reviewers, creating dedicated funding streams, and adopting novel approaches to decision-making.

#### Masking the applicant track record and allocating a ‘Golden ticket’ for reviewers

The NSF [45] and the Villum Foundation (VF) [59], successfully demonstrated that emitting researchers’ identities (i.e., institutional affiliations and publications) from applications gave the applicants ‘more confidence’ in proposing innovative but more risky ideas and the reviewers more ‘enjoyment’ in the review processes by allowing them to focus on the merit of the research and appreciate the novelty of ideas rather than the applicant track record (as per the notes from AR’s interview with Prof Thomas Sinkjær at the Villum Fonden (VF)). At the VF, this pilot scheme led to a third of grants being awarded to investigators who were < 40 years old; at the NSF, a similar pilot led to funding of proposals that would have otherwise been rejected. As an addition to their scheme, the VF also allocated their reviewers a ‘Golden ticket’ to nominate one proposal of their choice for funding regardless of whether it met official review criteria. This led to 31% of the grants being awarded in that cycle being chosen using the Golden ticket. The above evidence makes it clear that, as interventions to peer review, applicant anonymity and reviewer autonomy could promote the proposal of innovative research ideas and reduce the bias of review and funding panels against high-risk research.

#### Awarding grants to exceptional investigators

The National Institutes for Health (NIH) had launched their Director’s Innovator award in 2007 as a pilot of funding mechanisms tailored to supporting more investigators with high-risk high-impact research ideas. This was created to address long-standing concerns that the NIH’s review system was biased against unconventional research and (originally) offered ‘no-strings-attached’ grants to a small number of ‘exceptional’ researchers who could demonstrate a stellar track record and leadership in their field [60]. Today, the revamped ‘New Innovator’ award at the NIH is aimed at ‘early-stage investigators’ as part of the agency’s extensive High-Risk High-Reward Research Programme of Director’s Awards creating funding opportunities for innovative researchers at all career stages. An Early Independence Award is even available for ECRs wishing to bypass extensive postdoctoral training for career independence [61]. Prioritising high-risk research from ECRs, rather than leading investigators, therefore seems to be the new mechanism that the NIH employs to promote innovation and this in turn creates the long-term outcome of reducing burden for ECRs and giving them confidence to challenge convention and make use of their increasing exposure to interdisciplinary environments [62]. However, grant schemes such as the Director’s Pioneer Award [63] at the NIH that places emphasis on an ‘outstanding track record’ still exist, indicating that some conservatism will be practiced by funders to protect their investments and create competition for ECRs.

#### Using the Delphi process to promote innovation and impact

Two studies demonstrated that the Delphi process (explained in 38) can be used as an effective alternative application scoring mechanism for making funding decisions. The National Cancer Institute (NCI) used a modified Delphi approach to select clinical sites for the infamous American Stop Smoking Intervention Study (ASSIST) in the US; this involved convening an informal panel of decision-makers and iteratively requesting feedback on applications in the form of questionnaires and interviews as to whether they met key criteria for site inclusion into the study (e.g., smoking prevalence in the region) [64]. The significant benefit the ASSIST study had on smoking rates across the national study sites was evidence of the success of the Delphi model as a novel approach to decision-making. Indeed, panel members reported finding this method clear to follow and NCI programme managers added that it circumvented the political pressures and technical complexities they normally deal with during site selection. The Delphi process also worked for Cancer Council New South Wales in Australia, who identified a public need for prioritisation of innovative research into pancreatic cancer (a rare but devastating disease). The funders reported the process contributing to more balanced funding decisions by encouraging panel experts to ‘think outside the box’ and removing from panels confounding social factors such as seniority and ‘group think’ [65]. The process also made it easier to secure reviewers, which may go towards explaining its subsequent long-term implementation for the New South Wales Pancreatic Cancer Network Strategic Research Partnership Grant scheme [65]. With the advantages of greater accessibility and expediency, this application scoring mechanism may therefore add most value to decision-making in the more niche research areas like pancreatic cancer, where well-balanced critique of research ideas by a diverse panel of experts could promote funding of innovative solutions to difficult problems.

### CMOC 4 – selection and accountability of reviewers

The documented need for funders to increase their reviewer pools both in reviewer numbers and expertise to enhance funding decisions [66] has been associated with interventions in the literature aimed at using bibliometrics to identify experts and improving the accountability and impartiality of reviewers’ assessments of proposals.

#### Using bibliometrics to select reviewers

Abramo and colleagues [27] conducted a ‘top-down’ bibliometric screen of 1545 reviewers publicly registered with the Sardinian regional Ministry of Education, Universities and Research (MIUR) to determine the competency, scientific activity, and disciplinary coverage of candidates. From their statistical data, a worrying picture of regional grant review emerged whereby reviewer selection appeared to be highly subjective and ‘overinternalised’. Major disciplines such as Medicine lacked adequate reviewer coverage and a quarter of reviewers who were registered with MIUR turned out to be minimally or no longer active in research. The authors then investigated whether a reviewer registry created from reviewers’ bibliometric data could help improve (and objectify) the selection and management of peer reviewers and address gaps in expertise. Although this was a simulated intervention and the bibliometric selection of reviewers was not used outside of this study, its short-term outcome was compelling evidence that reviewer pools, and even large reviewer registries, can suffer from inadequate disciplinary coverage if reviewer selection and monitoring is inadequate. Furthermore, this suggests that bibliometric data can also be used to improve existing or create multidisciplinary reviewer registries by helping find active research experts for busy or underrepresented disciplines.

Another organisation in Italy called the Italian Cancer Network’s Training through Research Application Italian Initiative (TRAIN) evaluated the feasibility of using a semi-automatic reviewer selection tool, which made choices based on bibliometric indicators of expertise including citation indices, PMIDs, MeSH descriptors and qualifiers [28]. The tool led to 162 review candidates to be discarded and 205 to be accepted by TRAIN to assess fellowship applications in biomedical and health research. Programme officers praised the tool for the time and cost it saved them by removing the need to manually rank reviewers. However, 45 reviewers refused to participate in the scheme which suggests they reacted negatively to this concept – likely as it required candidates to provide PMIDs of publications as proof of expertise which can be seen as creating potential for performance bias.

A simulation study by the Slovenian Research Agency (SRA) suggested that bibliometric indicators can also be used to ‘control for conflict of interest’ in peer review by correlating reviewer scores with applicants’ bibliometric data to determine ‘objectivity’ of reviewer critiques and hence the fairness of funding decisions [30]. In 2008, this approach led to a 65% funding rate at the SRA; however, it should be noted that in a prior funding call the agency achieved similar success rates by implementing what they referred to as a ‘sound international peer-review system with minimised conflict of interest influence’ – perhaps indicating that although bibliometrics may enhance peer review, they should not completely replace the traditional system and that following the good review practices of others should be considered. This especially applies to countries that lack Slovenia’s centralised system of bibliometric data on all researchers.

#### Publishing reviewer identities, diversifying review panels and monitoring the impartiality of review

The Association of Medical Research Charities (AMRC) promotes good peer review practice across member charities in the UK by requiring they uphold core principles of ‘accountability’, ‘balance’, ‘independent decision-making’, ‘rotation of scientific advisors’ and ‘impartiality’. Following a 2011 audit of 114 member charities [67], the AMRC identified lack of transparency and impartiality in the peer review practices of some charities and provided them with tailored recommendations for interventions that would need to be implemented to pass the next audit. These included: sharing research strategies and peer review policy with researchers; publicising reviewer identities and funding rates; preventing conflict of interest; arranging independent site reviews; and diversifying review panels by recruiting multidisciplinary experts and experts from overseas). The subsequent audit [68] provided evidence of the long-term outcome of the AMRC’s recommendations, with charities implementing stronger conflict of interest policies, independent monitoring of review practices for impartiality, and transparency of peer review practices and reviewer identities.

### CMOC 5 – allocation of reviewers to proposals

Studies aiming to improve the matching of reviewers to proposals explored whether increasing the number of reviewers per proposal and involving applicants in the review process would work for stakeholders to increase reliability and quality of reviews.

#### Using bibliometrics to assign reviewers to proposals

The semi-automatic reviewer selection tool piloted by the Italian Cancer Network (described in CMOC 4) was evaluated for its ability to match bibliometric indicators of reviewer expertise with the keywords in proposals [28]. This worked well insofar that each reviewer was assigned at least one proposal (indicating the tool was fed sufficient data) and that a great deal of administrative time was saved (although users of the tool could still have final say over the tool’s reviewer assignments). The tool’s web-based interface also enabled accessibility from multiple devices. However, no evidence was given on the quality or interrater agreement of reviews resulting from these bibliometric matches and, as such, no measurable effect of the tool on reviewer reliability can be determined.

#### Using a tailored algorithm to match reviewers to proposals

The Slovak Research Development Agency (SRDA) attempted to use a network flow theory algorithm to assign proposals to reviewers, which worked by incorporating the funder’s own guidelines into the algorithm (for instance, the number of reviewers per proposal, how many external or internal, and the restrictions on memberships) [29]. Piloting of this algorithm by the Council of Natural Sciences resulted in the ‘successful and balanced’ matching of 83 proposals with reviewers without any conflict of interest. However, again there was no data on the resulting quality of reviews and the fate of this reviewer selection algorithm beyond this experiment remains unknown.

The Australian Research Council (ARC) carried out a retrospective study of the peer review processes used to fund national research to identify, where found, the major challenges to achieving reliability [69]. The issue was found to lie in external review practices, based on which the ARC then made recommendations to a) scrap researcher nominations of reviewers; b) assign more reviewers per proposal; and c) improve interrater agreement by assigning more proposals to reviewers. These recommendations act as long-term outcome evidence of the benefit to funders of considering not only the “who reviews?” but also, the “*how many* reviews?” questions of review management. However, it must also be considered while the recommendation to assign many proposals to a small pool of reviewers can benefit the review process, it can exacerbate reviewer fatigue (see CMOC 9 – unnecessary burden for reviewers section).

#### Assigning more than two reviewers per proposal

To determine the ‘ideal’ number of reviewers for a single application, the NIH ran a simulation experiment on > 80,0000 applications, aiming to achieve the highest possible interrater agreement [34]. Based on sample size analysis, the ideal number of reviewers per proposal was found to be four. However, the authors admitted that even implementing this agency-wide would be difficult, not to mention that it sets somewhat of an unrealistic goal for smaller funders and charities, who may already struggle to find enough reviewers to assign two per application. Perhaps a realistic compromise would be allocating, where possible, at least three reviewers to each proposal and/or to sure that the expertise of reviewers is best placed or overlaps multiple research areas (epistemically and technically). Attempting this is worthwhile as, according to the NIH, assigning more than two reviewers per proposal may help reliably score the more contentious/risky applications; as such, this intervention may be of particular interest to funders wishing to fund more innovative research (CMOC 3).

As a similar approach, researchers at McGill University conducted a study to demonstrate that the traditional two-reviewer system introduces a significant element of chance into decision-making [35]. The authors showed that assigning 11 reviewers to a single clinical research application using an independent ranking method prevented outlier reviews that can skew final application scores. Whether traditional review criteria were still used, however, was not mentioned in the study. While the short-term outcome of the pilot for the study is clear – a more reliable review process– it warrants considering how managing such a high number of reviewers may impact the funder it terms of administrative time and costs. Evidence of any organisation implementing that many reviewers was not found.

#### Involving applicants in the peer review process

A proposed solution to the issue of reviewer selection was explored by the NSF Civil, Mechanical and Manufacturing Innovation Division, who ran a pilot involving applicants in the review process to reduce the strain on the agency’s already stretched merit-review system. In the pilot, applicants were each allocated seven competing proposals to rank from best to worst [32]. The scoring system prevented applicants from ‘downgrading’ competing proposals and bonus scores were promised to researchers on their own applications if their reviews were honest and aligned to others’. This approach reportedly created a ‘captive and highly motivated’ pool of reviewers that required little time and effort from programme officers and resulted in reviews that were of similar quality to those provided by external experts. However, how the quality of reviews was evaluated beyond interrater agreement is information that was unfortunately lacking from the study. Moreover, some within the NSF expressed concerns that putting emphasis on group consensus in peer review could discourage innovative research ideas (CMOC 3).

### CMOC 6 – quality and reliability of peer review

Four interventions were implemented to improve the reliability of grant review by: training reviewers; employing independent review panels; and simplifying scoring systems.

#### Training reviewers to improve interrater reliability

The British Medical Journal (BMJ) published a randomised controlled trial (RCT) of the effect of reviewer training on the quality of peer review for publication [21]. This was included here as the need for more RCTs in grant review research and in particular better training of reviewers has been voiced by others [33, 70, 71]. The BMJ trial showed that the efficacy of training may depend on its modality, as reviewers who were randomised into receiving training via a self-taught electronic package, and not a taught workshop, showed significant improvement in review quality (identifying more errors in manuscripts and rejecting more manuscripts for publication). However, this outcome did not appear to be long-term, suggesting that repeated training sessions or a longer course may lead to better consolidation of learned skills than a single taught or self-taught resource. Indeed, since publication of this trial, Sattler et al. [33] conducted the first RCT of the effect of reviewer training on interrater reliability of grant review scores (although these were scores of proposal summaries, rather than full proposals). The findings revealed that even a brief training programme in the form of an 11-min video led to a significant increase in the reliability of review, as measured by scoring accuracy, interrater scores, and time spent studying review criteria [33]. Moreover, this improvement was observed for novice and experienced reviewers, suggesting that everyone may benefit from better understanding of the grant review rating system. Scarcity of evidence makes determining the long-term outcome of grant review training (e.g., implementation beyond studies) difficult and more investigation is needed into determining what aspects of grant scoring reviewer training should focus on (e.g., defining review criteria or appropriately using rating scales).

#### Employing two independent review panels

The National Health and Medical Research Council (NHMRC) revealed using an RCT that using two independent review panels to score applications for the Early Career Fellowships scheme improves interrater agreement and the reliability of funding decisions – as measured by agreement of panel members and the impact of decisions [36]. An added benefit of this review system was that in enabled reviewers to put less emphasis on track record, giving a good example of how changes in one part of the review process may unintentionally affect another. In this case, the effect can be seen as positive as it discourages bias against ECRs and innovative research (CMOC 3) and promotes a research culture that is less reliant on performance metrics.

#### Simplifying scoring systems

The University of Texas evaluated the reliability of a Grant Proposal Rating Form (GPRF) for assessing proposals at the Hogg Foundation (a major funder of mental health research in the US) [37]. The findings suggested simplifying the scoring system by replacing ‘scale-based’ scores (e.g., ‘not at all’ (0), ‘somewhat’ [1] and ‘definitely’) with dichotomous scores (e.g., ‘yes’ [1] and ‘no’ (0)) leads to an equally reliable, but simpler, review process.

As another example, the Academy of Finland showed in an observational study [72] that in situations where two experts held highly opposing opinions on a proposal, averaging their scores achieved consensus, and that collating reviews in one report would allow reviewers to combine their individual areas of expertise and bridge any epistemological differences. The authors argued that this highlights the importance of diversifying review panels to achieve a multidisciplinary group of experts for a more reliable review process (also discussed in CMOC 4 – selection and accountability of reviewers section).

### CMOC 7 – patient and public involvement in funding decisions

Two publications evaluated mechanisms of improving public engagement in health research through interventions focusing on applicant and reviewer training and involving public members, patients, and health advocates in funding decision processes.

#### Promoting community engagement of research through applicant and reviewer training

Piloting of a community engagement and peer review framework at the NIH [73], which involved holding work groups of public members, academic experts, patient representatives and reviewers to explore the perceived lack of emphasis on community health priorities in the agency’s funding decisions. Group discussions and analysis of previous NIH reports identified the need to better educate ECRs on community engagement of research and provide clearer guidelines on community engagement in peer review and funding decisions. Following these internal interventions, the NIH was able to redefine ‘community engagement’, identify strategies for researcher training and develop guidance criteria for reviewers. Long-term these interventions were developed into a community engagement framework.

#### Involving public members, patients, and health advocates in decision-making

The University of Wisconsin–Madison Institute for Clinical and Translational Research (ICTR) demonstrated in an NIH-funded pilot that employing an External Community Review Committee (ECRC) to re-review, from a community standpoint, proposals already reviewed by experts increases the community relevance of funding decisions while ensuring their scientific merit [102]. As part of the intervention, members of the ECRC were subjected to an intensive training course on peer review and the programme’s mission – which we think provides an interesting example of an “intervention within an intervention”. Importantly, as a result of the ECRC’s input into the review process, nine projects were selected for funding that would have not been selected by the traditional method – further demonstrating the need to incorporate community engagement into review criteria. As additional outcomes, the programme generated six externally funded grants and seven peer-reviewed publications. A similar intervention at the Patient-Centered Outcomes Research Institute (PCORI) engaged scientists and other stakeholders (members of the public and patients) in a two-tier review system, which revealed that inclusion of wider stakeholder groups in decision-making can improve both the translational and social relevance of research [74]. The success of the funding outcomes led to the intervention being modified into a one-phase review process that gave all stakeholders equal authority over review criteria and implemented in further funding calls at the institute.

The benefit of a two-tier review system was also reported by the Michigan Institute for Clinical and Health Research (MICHR) [75], who showed that subjecting proposals to a two-tier review process involving a Scientific Review Committee and a Community Engagement Coordinating Council over time led to funding decisions that became less based on the research budget and more on review scores for scientific merit and community engagement of research. Community engagement scores were based on criteria of ‘involvement, priority, and benefit’, which is an easily transferable model to adopt should other funders wish to follow the MICHR’s example.

The National Breast Cancer Coalition also piloted a Patient and Public Involvement (PPI)-based two-tier review system [76] where the traditional expert-led round of review was followed by review by a smaller panel of research ‘consumers’, represented by survivors of breast cancer and patient advocates. In addition, consumers were also included in funding committees and this consumer input into decision-making resulted in 15% of applications being short-listed for funding. Most stakeholders reacted positively to this intervention (84% of scientists and 98% of consumers), although some experts were concerned that the consumers’ lack of experience in peer review could affect the technical merit of funded research. Presumably, this can be avoided if consumers can only consider proposals that fare well in the first round of review and are, therefore, scientifically sound.

### CMOC 8 – unnecessary burden for applicants

Six interventions addressed areas where grant writing and review was shown to pose unnecessary administrative burden for applicants [9, 38]. These involved shortening applications to limit technical detail, improving feedback for applicants, open access to reviewer comments, improving applicants’ grant writing skills, educating applicants on peer review and decision-making, and promoting applications from new investigators.

#### Shortening applications and limiting technical detail

An observational study led by the Australian Centre for Health Services Innovation (AusHSI) investigated the effect of a ‘streamlined’ funding process on applicant burden and funding rates [43]. Cutting down applications to a 1200-word limit and focussing on the research question, methodology, budget, healthcare partnerships and potential impact of the research resulted in a 50% increase in application shortlisting and a 23% increase in interview invitations. Remarkably, applicants reported that it took them on average only seven days to prepare proposals, suggesting that a lot of the effort normally involved in submitting full research proposals to funders (which can be > 100 pages and take weeks or even months to prepare) may be unnecessary.

A long-term outcome of this intervention was demonstrated by the NIH, who from 2010 implemented a shorter version of their most popular but longest application, the Research Project Grant (R01), following a decision to reduce the ‘research plan’ section of the application from 25 to 12 pages [44]. Lawrence Tabak, who is now acting director of the agency, defended this decision by arguing that shorter applicants will force both researchers and reviewers to focus on the big picture and not be distracted by technicalities (e.g., fine details of established methods that say nothing of the impact of the research). The ‘short well-defined proposal’ strategy is already supported by other funders such as the Wellcome Trust [44], which suggests that shortening applications may be one intervention that funders should consider applying universally to reduce burden without any negative outcomes for stakeholders. The Molecular and Cellular Biosciences division of the NSF went a step further and shortened proposals to a two-page synopsis of the ‘central research idea’ in an experimental scheme called the ‘Big Pitch’ [45]. While the findings of this scheme were not released, its long-term outcome was implementation of the synopsis format in subsequent NSF funding rounds (which was eventually increased to four pages at the request of reviewers). The reviewer feedback here should be noted by those who may worry that shortening applications will cost reviewers the ability to assess their scientific merit, since in such a case, reviewers would have the power to request more information from the funder or applicants.

Finally, the success of the 2017 experiment of the Villum Foundation’s to ‘fund ideas and not pedigree’ (described in CMOC 3) was not only owed to the anonymisation of candidates submitting innovative proposals (who may have been ECRs), but also their shortened format of a three-page summary as part of the funder’s strategy to focus reviewers on the impact of research and not the track record of the researcher [59]. However, the outcome of the experiment and the feedback of participants did not include an in-depth insight into how, or how much, the shorter applications contributed to funding decisions.

#### Improving feedback for applicants

Researchers applying for funding to the Alberta Heritage Foundation for Medical Research (AHFMR) reportedly routinely requested more feedback from reviewers, which the AHFMR addressed in their pilot study [77] on the benefit of ‘ProGrid’ software to decision-making (described CMOC 10). Providing applicants with ‘additional and more meaningful feedback’ was one of the primary outcomes and was achieved by the ProGrid decision tool in the form of an automated summary feedback report. While the quality of the feedback was not assessed in the study, it was well-received by applicants who then made suggestions as to how to improve it.

The National Taiwan University of Science and Technology demonstrated a positive correlation between the quality of applications and the quantity of feedback provided to applicants in an experimental scheme in educational research, where applicants received three rounds of online peer feedback [78]. Significant improvement in application quality was observed following the first-to-second round, but not the second-to-third round, of feedback which according to the authors suggests the importance of helping researchers understand proposal criteria early in the application process. Interestingly, the above was echoed by RAND Europe [79] who recommended in a consultation panel on peer review practices convened by the Canadian Institutes of Health Research (CIHR) that researchers should receive feedback on applications as soon as they pass the triage stage, before peer review and funding committee decisions [80]. Similarly, an NIH pilot of a ‘streamlined’ funding cycle showed that shortening the review window and convening funding committees earlier gave applicants more time to address feedback on their proposals (which included proposal scores, reviewer comments and funding committee summaries) before making resubmissions [44]. The resubmission deadline was in turn extended by three weeks so that applicants did not have to wait until the next funding cycle to resubmit their proposals. The streamlined funding cycle was later implemented agency-wide [81] and, together with the above examples, serves as evidence that the ‘quality’ of feedback for applicants, and hence the quality of applications, may be a question of when the feedback is given and not just what the feedback contains.

#### Open access to peer review

The international Digital Humanities Conference implemented a set of reforms in 2012 to facilitate evaluation and exchange of peer review [82]. One measure was to make reviewers’ comments on research presentations/proposals accessible to other reviewers. This encouraged more ‘thoughtful and constructive critique’ of proposals and the reviewers to modify their original comments if others found them unfair. The outcome of these changes was a cross-community, rather than a top-down, review structure that benefited from increased accountability and transparency (discussed in CMOC 4 – selection and accountability of reviewers). Today, open access to peer reviewed research is a mainstay of some interdisciplinary spaces and includes examples like the Digital Scholarship libraries and the arXiv and bioRxiv pre-print archives for biomedical research output. However, beyond encouraging an open research culture, it is uncertain whether organisations in fields like health are likely to take up an open approach to peer review for making funding decisions and the consequences this may have for all stakeholders involved.

#### Improving applicants’ grant writing skills

An example of how funders can help researchers submit better proposals is the Venture Research Unit set up by British Petroleum (BP) in the late ‘80s to fund more ‘transformative’ ideas research and support researchers through the application process [46]. This was achieved through outreach activities, such as presentations at universities and workshops to help applicants write proposals that aligned with BP’s research priorities. In outcome, a total of £20 million was awarded to 30 researchers across Europe and North America through the scheme. For funders not offering such schemes, this example should highlight the potential effectiveness that their own input into the application process and the resulting improved qualities of proposals can have on increasing return on investment. More recently, the NSF made a plan to offer more outreach to HEI researchers and increase proposal success rates by publicising institutional submission and success rates; discussing with researchers their institutions’ submission policies; developing mentoring programs; encouraging researcher networking; educating researchers on the funding process; and helping researchers prepare stronger proposals. The report on this plan demonstrates the different ways in which funders can support applicants with grant writing to create a bigger impact (e.g., encouraging an open research culture). Supporting applicants in writing proposals that are highly relevant to a funder, (which may incidentally increase odds of success) eases unnecessary burden on applicants by saving them spending time writing an application that does not meet the requirements of the research programme or funding call.

#### Educating applicants on the research funding processes

Lack of HEI-level support with grant writing was highlighted and investigated by three research groups.

Georgetown University Medical Center designed an internal student-run research funding programme that trained PhD students on writing biomedical proposals and allowed them to engage in peer review [83]. The scheme achieved a high success rate of 46% and students reported being left with a better understanding of how research funding works and valuable experience of writing and reviewing grants. The School of Medicine at the University of Wisconsin developed a three-round educational research programme to improve the quality of education and reduce the burden of grant writing for faculty staff [84]. The outcomes of the programme included 28 internally funded proposals, a 200% increase in external investment into the medical school, two externally funded grants, publications, and dissemination of research across national and local meetings.

The National Taiwan University of Science and Technology designed a four-month online master’s course in Educational Research Methods to educate applicants in research methods as part of an experimental intervention to enhance the overall quality of grant writing, described earlier in this CMOC (see Improving feedback for applicants section) [78]. While the overall outcome of the scheme was successful in demonstrating the importance of early peer feedback to the improvement of proposal quality (which was already captured earlier), it is unclear how much the course contributed to this improvement.

#### Promoting applications from new investigators

The NIH created a ‘New Investigator (NI) Award’ to in response to a historical increase in the average age (> 40 years) at which investigators received their first NIH grant [85]. Adding a ‘new investigator’ tick box to their Research Project Grant (R01) application form, enabled reviewers to focus on the novelty of the research and the quality of the research training environment. A pilot round of NI submissions revealed success rates that were similar to applications from established investigators (around 19%). Funding committees later made the decision to stop ‘streamlining’ NI applications (i.e., limiting discussions to the top 50%) and extended the deadline for resubmissions to following funding cycles. The NI grant remains in place today and is now accompanied by the ‘Early Stage Investigator Award’ for researchers who graduated within the past 10 years and the ‘Pathway to Independence Award’ (a mentoring scheme to prepare post-doctoral researchers for an independent career) – which are more focussed on ECRs than researchers not holding prior NIH grants [86].

### CMOC 9 – unnecessary burden for reviewers

Interventions to address reviewer burden involved optimising internal review structures, virtual panels, rotating reviewers, and application triage.

#### Optimising review structures

A six-year period of NIH reform saw a major restructuring of peer review structures and processes [81]. Redistribution of 5–6 basic science sections from individual review groups to larger study sections covering both basic and clinical research alleviated some of the pressure on review panels and monitoring was put in place to ensure that reviewers were matched to applications by expertise. Shorter R01 applications (see CMOC 8 – unnecessary burden for applicants) also meant more applications could be assigned to a single reviewer. The above can be used as an example for other organisations that interventions to improve the application and peer review process may require periodic re-evaluation of existing review structures.

#### Virtual panels

Further to the above, the NIH announced their vision of replacing in-person review sessions with virtual panels and to let reviewers serve shorter terms on panels or take breaks from study sections [44]. The importance of reviewers rotation was also echoed by the AMRC, who require reviewers who have served on panels for six consecutive years to step down for three years before returning [67]. When stepping down, these experts should be replaced by ‘ad hoc’ reviewers so that they can bring fresh insight into the process. A 2011 AMRC audit revealed that many (particularly small) charities lacked a reviewer rotation policy due to high reviewer rejection rates. For these charities, the recommendation of the AMRC was to seek reviewers with expertise in overlapping fields.

Other funders reported plans for, or positive outcomes in reducing reviewer burden using virtual panels. The NSF set out a plan to increase investment into virtual technology so that a significant portion of their review processes would use virtual panels as standard practice [87]. They also outlined a plan to increase the size of review panels to minimise fatigue for individual reviewers. The Marie Sklodowska-Curie Actions under EU’s Seventh Framework Programme for Research showed that an additional benefit of remote review (which they conducted on 24,897 scientific proposals between 2007 and 2013) is a significant reduction in review panel costs that does not affect interrater reliability across panels and grant calls [88]. As a result, this approach remains in place at the organisation today. Similarly, for the American Institute of Biological Sciences (AIBS), use of teleconference meetings made convening funding committees easier and showed that efficacy of decision-making did not depend on social settings [40]. When compared to in-person meetings over a two-year period, teleconference meetings at AIBS showed no effect on overall application scores, nor did the remote settings contribute to any contentiousness or outlier bias in discussions.

#### Application triage

Following controversial changes that the CIHR made to their peer review processes, an international panel convened by them and led by RAND Europe made recommendations on their peer review practices [79]. These included setting up realistic triage to evaluate application demand against the funding available with a view to reject applications with a low chance of meeting funding criteria/thresholds. Recommendations for continuous enhancements of peer review included the CIHR clarifying their expectations regarding feedback to support reviewers and applicants in providing and responding to feedback on applications that have passed the triage stage.

### CMOC 10 – unnecessary burden for funders

Interventions to reduce funder burden included controlling application demand, implementing peer review within researchers’ host institutions, virtual panels and automation, expanding the reviewer pool, using decision models, and streamlining the funding process.

#### Controlling application demand

In 2000, a 50% increase in R01 applications prompted the NIH to consider measures of reducing administrative burden, namely introducing limits on R01 re-submissions to one per applicant per cycle and marking the weakest proposals as ‘not recommended for re-submission’ – a policy that remains in place today [81]. The NSF also placed quotas on new applications to three ‘preliminary proposals’ per institution and made full submissions ‘invitation-only’ [42]. The invitations in turn were limited to two per institution per year as a measure to reduce staff burden and encourage institutions to triage their own applications (see Introducing internal peer review section). This submission policy was not well-received by researchers who saw it as the funder shifting the burden of peer review onto the HEIs and increasing internal competition [42]; nevertheless, the NSF has further tightened this quota to a single proposal per institute for some of its programmes [89]. The University of Wisconsin also addressed funder burden by limiting submissions per investigator; moreover, they recommended introducing a cooling-off period between submission rounds. This was evaluated through a simulation of a funding cycle, which aimed to show how altering the funding strategy would impact on applicant behaviour, the number of submissions, the efficacy of the funding processes and the funder burden [90]. Based on the results, the authors suggested that limiting submissions may be more effective than allowing applicants to adopt a ‘multiple proposals strategy’ to increase their chances of success.

#### Introducing internal peer review

The Department of Psychiatry at the University of Pittsburgh introduced an internal peer review system where applications to the NIH would be scored by faculty peers before submission [91]. While the impact of this system on application success rates was not published, there was a reported increase in interdisciplinary publication output as a result. The reactions of the applicants to the increase in competition the internal review system would have presumably created were not published.

#### Using virtual panels and automation

The NSF’s plan to cut their yearly expenditure on conducting merit review involved standardising virtual panels across funding calls (described earlier in this CMOC). Following a calculation of the cost of in-person review panels in 2011 ($38 million a year), a pilot replacing these meetings with virtual meetings was found to save the NSF $5 million a year. A plan was then set out to conduct at least 33% of review panels virtually, while continuing investment into virtual technology (such as desktop equipment and cloud-based software) [87]. The same report included a plan of the NSF to evaluate the efficacy of an ‘automated proposal compliance checking system’, with an aim to simplify application processing and redirect valuable administrative capacity to the merit review process.

#### Enhancing the reviewer pool

The University of Wisconsin’s simulation of a funding cycle (see Controlling application demand section) showed that increasing the reviewer pool, allocating more reviewers per proposal, and using group consensus could not only benefit reviewers but also reduce burden for the funder [90]. However, according to the simulation, this approach would only work in conditions where funding demand is also reduced (e.g., through submission quotas), raising the question of its feasibility in the current climate.

#### Using decision models

The AHFMR piloted a researcher training scheme where funding decisions were supported by a specialised software called ProGrid [77]. Criteria for the automatic evaluation of applications were based on a ‘performance matrix’ of academic record, research experience, reference letters, supervisor background, resources, the trainee’s role in the project, the overall merit of the proposal, and the training environment. The software integrated reviewers’ scores and generated a summary report including the relative position of each applicant on the performance grid, specific reviewer comments, customised reports, comparison/average ratings, and the final ratings. As such, using this data output represents an objective, consistent and comprehensive method of decision-making; indeed, the AHFMR then continued to optimise and implement this software in other trainee programmes and reported that it simplified proposal discussions at funding committees and shortened the lengths of meetings. Similarly, the US Agricultural and Food Research Council (AFRC) showed that a decision software called Teamworker can support fairer discussions at funding committees by: calculating committee scores and revealing any inconsistencies, facilitating decision-making, encouraging debate, and controlling for bias [92]. This decision model received positive feedback from both the AFRC and HEI researchers and showed additional benefit when repurposed for evaluating staff promotions.

The Spanish Ministries of Education and Sciences showed that statistically analysing the academic performance of applicants could help predict the likelihood of research success and inform decision-making [31]. Applying this to the Spanish State Research Agency’s (SRA) Ramón y Cajal fellowship scheme, the relationship between review outcomes and applicant CV data was modelled to determine the probability of an established investigator being accepted to the programme. It was found that the software was able to reliably identify which applicants were ‘good’ based exclusively on factors relating to research productivity and no other variables, such as age and gender (which are known biases). The recommendation of the SRA was to optimise these decision tools to support and simplify applicant shortlisting.

#### Streamlining the funding process

An observational study of an accelerated (two-month) funding process at the Australian Centre Health Services Innovation (AusHSI) showed that streamlining funding cycles can reduce cost and administrative burden for funders [43]. Interviews were conducted within ten days of application shortlisting and applicants were notified of interview outcomes within two weeks (eight weeks after the submission deadline). This successful outcome was attributed to shorter applications (limited to 1200 words), more focussed application content (the research question, methods, budget, expected impact, and healthcare partnerships), and a simplified application review system of ‘reject’, ‘revise’ or ‘accept to interview’. Interestingly, there was also a reported reduced emphasis on the applicant’s track record which may represent an unintended but positive consequence for researchers (especially ECRs) (discussed in CMOC 3 – funding and support for innovative research section).

### Searches of funder websites

The results of funder website searches (see Methods section) are provided in Supplementary file, Update on recent funder interventions. Evidence extracted from 36 web sources covers relevant funder announcements made between 2017 and 2020, as well as the most recently published AMRC audit [68].

The results provide a list of interventions in peer review and new funding schemes that have been implemented, are currently undergoing research, or are planned for the near future. The global funders involved in these are listed and interventions are ordered according to ‘popularity’ (i.e., number of funders featured). The interventions were also categorised into ‘impact-oriented changes to funding strategy’ (i.e., changes that aim to benefit stakeholders outside academia), ‘organisation-level changes to funders’ (i.e., changes to the funder’s internal processes or structures (e.g., study sections) and ‘incremental changes to peer review and decision-making processes’ (i.e., changes to a specific part of the peer review process, as described previously [6]). The aim of this analysis was to capture how the priorities and practices of funders have changed since the literature included in the synthesis was published. However, as the scope of this work did not include a systematic screening of every funder website, the list was limited to the major international organisations cited in the publications and aim to offer only a ‘snapshot’ of recent funder interventions. As such, it must be noted that information from many small charities and private institutions will be missing from the analysis. To help account for this, we included some outcomes from the 2016) AMRC report on charities in the UK [68]. It is also important to note that this data does not reflect changes that have not been published by funders or that have occurred too recently to be evaluated.

The most popular impact-oriented intervention, implemented by nine global funders including the Q Exchange (Health Foundation over 4000 members) [93], was ‘promoting innovation in research’. The most popular organisation-level intervention, implemented by five funders, was ‘promoting co-funding of research’ and the most popular incremental change to PRDM, also implemented by five funders, was ‘modifying the composition of review and decision panels’. Information on funder uptake of other interventions, such as promoting ECRs, introducing new award schemes and monitoring peer review, is also provided (Supplementary file, Update on recent funder interventions). Of these, seven interventions were included as ongoing pilots or commissioned research. As an example, in 2020 the UKRI announced a goal to reduce unnecessary administrative burden across funding schemes and streamline the application process in a small-scale pilot [94]. The New Generation Thinkers 2020 pilot would evaluate a digital application form to the Arts and Humanities Research Council that would replace the standard pdf document format [95]. Around the same time, the Wellcome Trust commissioned three research teams to re-evaluate how they make funding decisions, which would include reviewing their assessment criteria and the end-to-end funding process [96].

## Discussion

The aim of this work was to determine which past interventions to peer review and decision-making have worked to improve the research funding process, how they worked, and for whom. We identified 50 interventions from the evidence base that for 17 international funders, 16 research institutions/organisations and 4 public authorities worked to enhance peer review by addressing what we identified to be 10 common themes: scientific, economic and social benefit of research; researcher career stability; funding of innovative research; selection and accountability of reviewers; allocation of reviewers to proposals; quality and reliability of review; PPI in research funding; and unnecessary burden on applicants, reviewers and funders. Interventions demonstrating long-term positive outcomes for stakeholders were training applicants in grant writing [83], publishing review scores [33], involving patients and health advocates in decision-making [74–76], introducing submission quotas [42, 91], shortening applications [43–45], incorporating (in selected calls) sandpits, randomisation, or Golden tickets to funding peer review processes [59, 64, 65], and virtual panels [41]. Overall, these interventions equipped applicants with better knowledge of the peer review process, reduced grant writing burden and the time taken to review full proposals, reduced interrater variability in review, increased the relevance and innovation of funded research, and reduced the costs of review panels.

The majority (65%) of the literature enabled extraction of contexts-mechanisms-outcomes (CMO) links for individual interventions implemented in the past by international stakeholders to improve peer review and decision-making. Configuration of these into broader themes representing the common issues in peer review revealed that most emphasis has so far been placed on reducing burden for applicants, reviewers, and funders. A further look at the high-level mechanisms addressing these issues revealed that five were focussed on enhancing decision-making (either through peer review or novel decision methods), and that four of five interventions promoting PPI in funding decisions involved restructuring peer review into a two-tier system of experts and other stakeholders (e.g., patients, advocates). It is of interest to us whether the pattern in these findings is representative of the wider work within this field of research.

We found that almost half of the interventions (47%) generated long-term outcomes for stakeholders and the research ecosystem (i.e., via changes to funding and review practices), while the remaining interventions (53%) generated short-term outcomes for funders or academia (i.e., in the form of direct study data or outcomes of pilot funding calls). Over half the literature (52%) could not be used for CMOC analysis as it described ‘theoretical’ interventions (e.g., opinion pieces) that have yet to be researched or implemented, from which outcomes could not be drawn. Although we expected this result since peer review is still more of a subject of debate rather than active research, it reinforces that more research and robust evidence (i.e., in the form of RCTs, prospective or feasibility studies) is needed to systematically evaluate and the efficacy of new interventions and their impact on the research ecosystem.

Frequency analysis of the different types of interventions described in the literature (both theoretical and evaluated) showed that in general interventions in peer review tend to focus on incremental changes to the overall funding process (e.g., shortening applications or allocating more reviewers per application). This finding supports the message of existing research [6] that suggest a realistic way forward may be to enhance specific mechanisms within the peer review process rather completely replace or attempt to standardise it. Moreover, combining incremental changes with innovative interventions may be the key to achieving long-term outcomes for stakeholders (e.g., enhancing the decision process by involving patients and the public, as previously shown [97]). However, it is important to note that although this work provides specific scenarios where interventions worked for certain funders or research contexts, evidence of any larger impact of these interventions on the research sphere remains difficult to obtain. Whether this is even possible to achieve through research needs acknowledging, since it is often hard to measure the true ‘scale’ of an impact, except to say that there has been one. Moreover, not all evidence of impact lies in research data. For instance, a glance at our CMOC analysis will reveal only one case where an audition system was used to match society-driven research ideas to relevant funders (CMOC 1). This makes it appear as if the impact and the generalisability of the intervention was limited. However, when considering that the stakeholders involved were 1) the government of Japan, 2) public and private sponsors of research and 3) the Japanese HEI system, and that the outcome of this intervention was a positive impact on the social relevance of research, the potential generalisability and wider impact of the intervention becomes clear.

Unsurprisingly, we observed that funders generated the highest publication output and were therefore the most active stakeholders in the community to promote or enact reforms in peer review (funders are held accountable for the research they choose to invest in using public or donor money so that it meets the many needs of society). To that end, our analysis of funder websites confirmed an ongoing global effort to continue re-evaluating and improving peer review and decision-making – as exemplified, for instance, by the NHMRC’s 2019 announcement to promote open access of peer review, virtual panels, reviewer autonomy and PPI in research [98]. However, a significant portion of the evidence is attributed to academic research into peer review, where simulation and feasibility studies were crucial in identifying areas for improvement such as training applicants in grant-writing and adopting an internal peer review system to improve submissions to funders.

Evidence of unintended consequences of some interventions highlight the need for funding organisations to work with other sectors (e.g., HEIs) as changes that worked for a funder created new or exacerbated existing issues for other stakeholders. An example of this was the NSF use of submission quotas to limit funding demand and the downstream effect of this on HEI departments and applicants (CMOC 10), which may have led to increased time and administrative effort from applicants who effectively go through at least two competitions for each proposal, first to be able to submit a research proposal to a funding organisation and then to obtain funds. As another example, the decision of the NIH to include ad hoc reviewers on review panels to minimise reviewer fatigue, diversify opinions and prevent undue influence of frequent reviewers (CMOC 9) ultimately resulted in an “*excessive ad hoc review service*”, which led the CSR to discontinue their policy of extending submission benefits to reviewers serving multiple times over a period of 18 months [99]. These examples highlight the complexity of how different elements in the funding process interact and, hence, the difficulty of finding solutions applicable to multiple organisations that benefit all stakeholders simultaneously. CMOC analysis also showed that multiple stakeholder mechanisms were relevant to more than one context (driver for change), highlighting that a single intervention can address different issues and equally affect multiple stakeholders.

It must be emphasised that making any clear recommendations from this work was challenging. While we were able to present 50 successful interventions, there was no evidence that any of these had any impact on peer review across the entire research funding sphere. As such, it remains impossible to recommend a ‘one-size-fits’ all approach that will work for all stakeholders in every research context. One intervention that could perhaps be universally applied by funders to reduce burden for stakeholders without adding new issues is shortening applications. However, one must remember that changing one aspect of a complex system (no matter how large) will not ‘fix’ the whole system. Our findings highlight the complexity of research funding processes and the common thread in these is that any real improvement – whether measured in longevity of outcomes, scale of impact, or generalisability to others – can only be achieved when the mechanisms (and stakeholders) of peer review and decision-making operate in harmony (e.g., when the research community works together to promote good principles of research and grant writing, or when funders show willingness to readily remove practices that don’t add value or create bias for certain researchers). We hope that the interventions to address common issues in peer review documented here will serve as further drivers for change in the field and provide guidelines for researchers and, in particular, other funders who may wish to incorporate some of these interventions into their current practices or future reforms. We encourage funders to continue re-evaluating new interventions in the ever-evolving research landscape and to facilitate further research (and promote a shared learning culture) by publishing the outcomes of any piloted or implemented interventions. Future research should focus on building an open database that captures different funder peer review processes to encourage transparency and accountability across the research community (similar to, for instance, the Platform for Responsible Editorial Policies for journals [100, 101]), and on creating or enhancing consortia of funders.

### Limitations

We acknowledge that this work will not have captured every successful intervention in peer review and decision-making as not all funder interventions are made public (reinforcing our message to funders to publish outcomes of pilots and implemented changes to practice). The generalisability of our findings may also be somewhat limited by the lack of quality assessment of the publications included and the interpretation of evidence, which was outside the scope of this study. Moreover, while we made the best endeavour to summarise and thematically sort a large body of evidence comprising highly complex and heterogenous interventions into a clear CMOC analysis, we acknowledge that the structure of this analysis (specifically, how we interpreted the ‘contexts’ and ‘outcomes’ of interventions) was based entirely on our own interpretation of the field and on examples of realist syntheses of interventions in more focussed areas such as health services research. As such, other researchers may draw different conclusions and arguments when reviewing the same literature. Finally, it is important to mention that the evidence presented here only covers interventions that have been carried out before the start of the Covid-19 pandemic, which had an unprecedented effect on the research landscape by requiring organisations to make rapid changes to maintain the funding process (e.g., by replacing in-person meetings with virtual panels) and to expedite the delivery and publication of health research. Capturing the outcome of these post-Covid changes warrants a separate study.

## Conclusions

To ensure that research funding is effective, fair, and relevant to the needs of society, mechanisms of decision-making, including peer review, must be subjected to continuous evaluation, innovation and public scrutiny. We have captured evidence of interventions that have shown success improving peer review and decision-making for a variety of stakeholders across diverse research contexts. According to what has been tried, a realistic approach to solving common issues in peer review and reducing burden for all stakeholders relies on making incremental changes to peer review processes and where possible, focus on innovation. However, it is important to consider that trade-offs are also likely to occur when making changes to complex funding mechanisms in an evolving research ecosystem of stakeholders with different priorities; as such, interventions can simultaneously generate benefit in one area while creating unintended burden in another.

## Supplementary Information

**Additional file 1.** Supplementary tables.

## Data Availability

Publications analysed during the current study are in the public domain and may be available from the corresponding author on reasonable request.

## Abbreviations

PRDM: Peer Review and Decision-Making
NIHR: National Institute for Health Research
CMOC: Context-Mechanism-Outcome Configuration
DARPA: Defense Advanced Research Projects Agency
CHSRF: Canadian Health Services Research Foundation
NERC: Natural Environment Research Council
EPSRC: Engineering and Physical Sciences Research Council
PCORI: Patient-Centered Outcomes Research Institute
AIBS: American Institute of Biological Sciences
CREST: Core Research for Evolutional Science and Technology Programme
RCUK: Research Councils UK
ECR: Early-Career Researchers
MIUR: Ministry of Education, Universities and Research
TRAIN: Training through Research Application Italian Initiative
ARC: Australian Research Council
SRDA: Slovak Research Development Agency
BMJ: British Medical Journal
RCT: Randomised Controlled Trial
GPRF: Grant Proposal Rating Form
PPI: Patient and Public Involvement
ICTR: Institute for Clinical and Translational Research
ECRC: External Community Review Committee
MICHR: Michigan Institute for Clinical and Health Research
CURES: Community-University Research Partnership
AusHSI: Australian Centre for Health Services Innovation
AHFMR: Alberta Heritage Foundation for Medical Research
CIHR: Canadian Institutes of Health Research
BP: British Petroleum
NI: New Investigator
SRA: Spanish State Research Agency’s
AFRC: US Agricultural and Food Research Council
HEI: Higher Education Institution
CSR: Center for Scientific Review
JST: Japan Science and Technology Agency
JSPS: Japan Society for the Promotion of Science
