# Supplementary material for: What works for peer review and decision-making in research funding: a realist synthesis
Source: Res Integr Peer Rev. 2022 Mar 4;7:2. doi: 10.1186/s41073-022-00120-2 (PMC8894828; doi:10.1186/s41073-022-00120-2)
Supplement: Supplementary file 1 — Additional file 1. Supplementary tables. [file 41073_2022_120_MOESM1_ESM.docx]

**Table of content for supplementary files**

1. Summary of findings of publications reviewed
2. Characterisation of publications
3. Types of interventions by frequency of use
4. Update on recent funder interventions
5. Search strategy in Medline (OVID)
6. Programme theory statements
7. **Summary of findings of publications reviewed (n=95)**

| **Author(s) & year** | **Study design** | ***Contexts*** | | | ***Mechanisms*** | | ***Outcomes (description)*** | ***Short-term* Long-term** outcome(s)*** |
| --- | --- | --- | --- | --- | --- | --- | --- | --- |
|  |  | **Research field** | **Location** | **Stakeholders involved** | **Intervention type** | **Intervention focus** |  |  |
| Abramo, D'Angelo and Viel (2013)[1] | Bibliometric analysis | Hard sciences | Italy | Higher Education Institute (HEI) and private sector researchers, Ministry of Education, Universities and Research (MIUR), external reviewers | Reviewer selection, bibliometrics | Use a ‘top-down' metrics-based screening approach to create a reviewer register | The study found that the disciplinary coverage and activity of the MIUR reviewer register was inadequate and unmonitored | * |
| Ahmed and Palermo (2010)[2] | Observational study | Health and clinical research | USA | National Institutes of Health (NIH, Institute of Medicine), NIH Director's Council of Public Representatives (COPR), wider research community, patients, the public | Promoting community engagement, applicant training in PRDM | Incorporate community engagement into the application requirements and provide training in community engagement awareness for researchers and reviewers | The COPR-developed community engagement and peer review framework was implemented across the NIH in 2008 | ** |
| Albanese et al (1998)[3] | Pilot | Health, clinical and biomedical research | USA | HEI medical faculty staff and students, NIH (National Cancer Institute), University of Wisconsin (UoW) Medical Foundation, Association of University Radiologists | Promoting educational research | Create a grant scheme for medical education research to improve the teaching quality, create new research opportunities and alleviate faculty staff burdens | A successful three-cycle pilot grant scheme led to funding of 26 new grants between 1995 and 1997, and continued collaboration between faculty and the medical foundation at UoW | ** |
| AMRC (2011)[4] | Audit | Health and clinical research | UK | Association of Medical Research Charities (AMRC; 114 charities in total) Parkinson's UK, Wellcome Trust, National Institute of Health Research (NIHR; Clinical Research Network Coordinating Centre), Neurological Alliance, Prostate Action, decision-makers, applicants | Applicant training in PRDM, reviewer accountability, open access, panel rotation, reviewer selection, modifying panel composition | Tailored AMRC recommendations included: education on the peer review process, publishing reviewer identities, grant panel rotation, broadening reviewer expertise, limiting external review to large grants, recruiting reviewers from abroad, conducting independent review of panel impartiality | The audit found that 18 charities failed to meet AMRC criteria; these were given recommendations to address for the next audit | ** |
| Andejeski et al (2002)[5] | Pilot | Health research (public and military) | USA | HEIs, National Breast Cancer Coalition, US Army Medical Research and Materiel Command, Department of Defence (DoD) | Patient and public involvement in PRDM | adopt a two-tier review approach to breast cancer research: an expert-led review of technical merit and an 'integrated' second review of impact by breast cancer advocates and survivors | A successful intervention, achieving interrater consistency between expert and public panels, positive feedback from all stakeholders and continued support from the DOD for implementation and transferability | ** |
| Avin (2014)[6] | Theoretical narrative | Hard sciences and biomedical research | USA | US/Western public and private research community, broad funding infrastructure, HEIs | Modifying funding allocation, secondary (applicant) allocation of funds, bibliometrics | Equally distribute funds to all applicants with a strong track record. Instruct awardees to further redistribute a portion of these funds at their discretion. Thus, remove the burden of PRDM but maintain funder reliability | N/A (concept only) |  |
| Avin (2019)[7] | Review | All research spheres | UK | Broad funding infrastructure, programme officers, policy-makers, decision-makers, applicants, reviewers | Random allocation of funds, modifying review criteria, modifying panel composition, shorter applications | Use graduated random allocation to fund proposals triaged into the scientifically 'middle-merit' (< top 10%) group. Reorganise panels by epistemic activity, limit application criteria and shorten proposals to include the applicant expertise and epistemic topic | N/A (concept only) |  |
| Barnett et al (2015)[8] | Observational study | Health research | Australia | Australian Centre for Health Services Innovation (AusHSI), Queensland Health Office of Health and Medical Research, HEI researchers, health and allied professionals | Shorter applications, simplifying PRDM, improving applicant feedback | Reduce application size (to 1200 words) and limit content to research question, methods, budget, partnerships and expected impact. Simplify scoring to 'reject', 'revise' and 'accept for interview' | Acceleration of the funding process to 2 months, shorter application times (~7 days), and faster notifications to applicants of outcome (within 2 weeks of interview) led to a 50% increase in shortlisted proposals and a 10% increase in funding | ** |
| Bhattacharjee (2012)[9] | Observational study | Biomedical research | USA | National Science Foundation (NSF, Molecular and Cellular Biosciences Division), HEIs, reviewers, applicants | Reviewer blinding, shorter applications | Shorten applications to a two-page synopsis of the research 'central idea' and anonymise applicant identities and affiliations. | Anonymised synopses led to reviewers funding proposals they wouldn't have otherwise funded by focussing on the novelty of the science and not the applicant track record. Four-page proposal synopses were applied to further NSF funding rounds. | ** |
| Bielski (2007)[10] | Observational study | Biomedical research | USA | NIH (Advisory Council to the Director Working Group), NIH stakeholders and leadership, applicants | Modifying funding allocation | (59 context-based interventions); two overarching suggestions to either focus NIH resources to 'top producers' or spread them across the NIH workforce | N/A (concept only) |  |
| Bollen et al (2014)[11] | Modelling | Hard sciences, biomedical and health research | USA/EU | NSF, NIH, European Research Council (ERC), HEIs | Modifying funding allocation, secondary (applicant) funding allocation | Maintain flow of funds through the research community by equal distribution of 'basic' grant and secondary awardee-directed distribution of fixed % of funds to researchers representing 'best value for money' | N/A (concept only) |  |
| Bonetta (2006)[12] | Pilot and analysis of funder reorganisation | Health and biomedical research | USA | NIH (Center for Scientific Review, Integrated Research Groups; IRGs), HEIs, clinical trial centres, professional societies, National Academy of Sciences, independent advisory groups, societies for basic sciences, applicants | Reviewer rotation, promoting early career researchers (ECRs), improving applicant feedback, promoting resubmissions, continuous improvement of funding structures and processes | Invite external and advisory reviewers on ad-hoc basis to encourage re-evaluation of review implementations and reduce reviewer fatigue. Facilitate ECR transition to primary investigator with a focus on training. Redistribute reviewer expertise around larger application groups. | Pilots in 2000-2006 led to IRG reorganisation in 2006-2008, involving a new Pathway to Independence Award Program, reviewer lenience towards ECRs submitting R01 applications, increasing ECR resubmission rates, removal of 5-6 basic science IRGs, and monitoring of reviewer expertise | ** |
| Bonetta (2008)[13] | Analysis of funder reorganisation | Health and biomedical research | USA | NIH (CSR), HEIs, clinical trial centres, professional societies, National Academy of Sciences, IRGs, societies for basic sciences, applicants | Shorter applications, reviewer incentives, simplifying PRDM, modifying review criteria, continuous improvement of funding structures and processes | Reduce application size by focusing on the 'bigger picture' rather than minute scientific details, limit resubmissions to strongest applications and simplify the review process | NIH R01 applications reduced from 25 to 12 pages and resubmission rates were decreased. Reviewer flexibility (term length, IRG) and virtual communication was encouraged from 2009. | ** |
| Boyack et al (2014)[14] | Bibliometric analysis | Health and biomedical research | USA | NIH (CSR) | Continuous improvement of funding structures | System-level visual mapping can be used by funders to better monitor and re-evaluate existing review structures | N/A (concept only) |  |
| Brumfiel (2005)[15] | Analysis of funder implementation | Hard sciences and engineering | USA | NSF, HEIs, applicants, reviewers | Introducing submission quotas | Restrict the number of submissions per institution and promote institution-level screening of application quality, thereby increasing funding rates | The NSF placed limits on application submissions per institution and set a requirement for HEIs to screen applications prior to submission, leading to higher success rates | ** |
| Cabezas-Clavijo et al (2013)[16] | Bibliometric analysis | Clinical research, hard sciences and engineering | Spain | Spanish Ministry of Science, National Administration of Public Education (ANEP), applicants | Bibliometrics, modelling decision-making | Use bibliometric indicators of applicant performance to complement peer review and better predict funding | N/A (concept only) |  |
| Canibano et al (2009)[17] | Modelling | Hard sciences, biomedical research, other spheres | Spain | Spanish Ministries of Education and Science, decision-makers, reviewers | Reviewer accountability, modelling decision-making | Use applicant CV data and modelling software to determine whether peer review outcomes can be facilitated using statistical tools | Applying this analysis of applicant data to the Ramón y Cajal Fellowship Programme was predicted to reduce funder costs and reviewer burden | * |
| Carpenter et al (2015)[18] | Retrospective analysis | Biomedical research | USA | American Institute for Biological Sciences, HEIs, decision-makers | Virtual panels, teleconferencing | Use teleconferencing instead of face-to-face meetings to diversify and ease the burden of decision panels | Replacing face-to-face meetings with teleconferencing in the PrX programme reduced funder cost and time burden, without altering the quality/effect of discussions | ** |
| Cechlarova et al (2013)[19] | Pilot | Clinical research, hard and social sciences, other spheres | Hungary, Slovakia | The Slovak Research and Development Agency, Council of Natural Sciences, HEIs, reviewers, applicants | Reviewer selection | Use a network flow theory algorithm to assign internal and external reviewers to applications, based on the funder's formulated guidelines | Applying the network algorithm to the 2011-2012 Council of Natural Sciences funding cycle led to a highly balanced assignment of reviewers to applications (under SRDA rules) that was free of any conflict of interest or lengthily personal negotiations | * |
| Chen & Tsai (2009)[20] | Observational study | Education | USA, Taiwan | HEIs, educational research experts, applicants | Applicant training in PRDM, promoting educational research, improving applicant feedback | Improve the quality of grant proposals by providing applicants with an online course in educational research methodology, as well as expert and peer feedback | Feedback was found to be most useful for applicants in the initial stages of preparing a grant proposal. | * |
| Cherfas (1990)[21] | Trial | Other sphere (agricultural) | USA | Agricultural and Food Research Council (AFRC), Ministry of Defense (MoD), decision-makers | Modelling decision-making | Use a software- and hardware-based statistical tool to facilitate fair discussions and effective decision-making by funding committees. | Trialling of the Teamworker decision-making tool for over a year enriched the decision-making process by providing precise and rapid analysis of information, fostering crucial debate and controlling for bias from a single committee member. Both the AFRC and the MoD gave the software a positive review. | ** |
| Clarke et al (2016)[22] | Trial | Health and clinical research | Australia | The National Health and Medical Research Council (NHMRC), HEIs, ECRs, reviewers | Modifying scoring of proposals, improving interrater reliability, promoting ECRs, modifying funding allocation | Increase reviewer reliability by implementing independent scoring to control for strong 'outlier' opinion in review panels and deprioritise applicant track record in decision-making. Remove the need for application budget assessment by implementing fixed budget fellowships for ECRs | Review of 60 ECR applications to the NHMRC by two independent grant review panels across four studies led to funding rates ranging 25-50%. | * |
| De Los Reyes & Wang (2012)[23] | Theoretical narrative | All research spheres | USA | All national funding organisations, decision makers | Modifying funding allocation | Allocate research budget based on the panel score: the higher the score of the proposal, the more of the requested budget is allocated. The applicant can then decide how to best utilise the awarded budget - to partially complete research objectives, revise the proposal or resubmit an application. | N/A (concept only) |  |
| Demicheli & Di Pietrantonj (2007)[24] | Review | All research spheres | Multiple countries | Broad funding infrastructure, reviewers | Reviewer blinding, improving interrater reliability | Different processes in peer review (screening or anonymising submissions, eliciting internal/external opinions and providing applicant feedback) affect its impact on the importance, relevance, usefulness, methodological and ethical soundness, completeness and accuracy of funded research | Overall, there is little empirical evidence that aspects of the review process ensure funded research quality: screening applications does not significantly impact review results; blinding of peer reviewers to applicants affects their assessment; reviewer agreement is high regardless of the way proposals are assigned | * |
| Dumanis et al (2013)[25] | Analysis of funder implementation | Biomedical research | USA | Georgetown University Medical Center (GUMC), Student Research Grants Program, HEIs, PhD students | Applicant training in PRDM | Recruit post-graduate students applying for grants to also review grants in the same focus area to increase knowledge about the grant funding process | The student-led research grant programme was implemented at GUMC for 3 years; it demonstrated overall increased knowledge on the grant application process among PhD students and reduction in their research burden | ** |
| Durso (1996)[26] | Theoretical narrative | Biomedical research | USA | NIH, University of California, reviewers, applicants | Modifying review criteria, promoting innovation | Modify grant review criteria to better identify and prioritise innovative research in applications | N/A (concept only) |  |
| Fang & Casadevall (2016)[27] | Theoretical narrative | Biomedical research | USA | NIH, intramural and extramural researchers, HEIs, private research industry, medical schools | Random allocation of funding, modifying funding allocation | Use a modified lottery approach for allocation of funds, whereby funding decisions are computer-generated and the size of the meritorious pool is adjusted according to the payline. Remove panels by offering feedback only to non-meritorious proposals, which would become eligible for resubmission in the next cycle. | N/A (concept only) |  |
| Fielder & Vinyard (1998)[28] | Theoretical narrative | Health research | UK | National research charities, Royal National Institute for the Blind (Prevention of Blindness Sub-Committee) | Open access, improving applicant feedback, eliminating cronyism in decision-making | Allow applicants to read the comments of external reviewers prior to funding panels and prevent committee chairpersons from applying for funds from the same charity | N/A (concept only) |  |
| Fleurence et al (2014)[29] | Observational study | Health research | USA | Healthcare charities, Patient-Centered Outcomes Research Institute (PCORI), research community, clinicians, patients, the public, reviewers | Patient and public involvement in review, improving interrater reliability | Include the input of patients, the public and the clinical and research community in the review process to reduce selection bias, better apply review criteria and improve study relevance (as these stakeholders will be implementing the study findings) | Positive feedback from reviewers, strong interrater agreement and improved critique during the pilot funding cycle led to implementation of this review approach by PCORI. | ** |
| Forsdyke (1991)[30] | Theoretical narrative | All research spheres | USA | Broad funding infrastructure, reviewers | Bibliometrics, modifying review criteria | Objectify peer review and make it less error-prone using a bicameral process, whereby the main criterion of scientific merit is replaced with an exclusive focus on the research budget and team track record | N/A (concept only) |  |
| National Science Foundation (2017)[31] | Analysis of funder implementation | Hard sciences and engineering | USA | Broad funding infrastructure, NSF, HEIs, decision-makers, reviewers | Virtual panels, reviewer selection and rotation, teleconferencing, applicant training in PRDM | Reduce travel costs and time burden with virtual reviewer panels and broaden the pool of qualified (ad hoc) reviewers using an automated compliance checking system. Increase outreach to specific HEIs to propagate best practice and improve grant success rates | Based on pilot data and recommendation, the NSF planned to invest into virtual meeting technologies, review applicant support, automate preliminary funding processes and manage demand | ** |
| Freireich (1990)[32] | Observational study | Health research | USA | NIH, national clinical cancer research, applicants | Reviewer proposal matching, promoting clinical research | Improve competition in funding of clinical research by assigning proposals to clinical (and not laboratory) research peers for review and identifying the unique requirements of clinical research | N/A (concept only) |  |
| Frodeman (2012)[33] | Theoretical narrative | Health and biomedical research, hard sciences and engineering | USA | NIH, NSF, Comparative Assessment of Peer Review (six global agencies), | Reviewer accountability, patient and public involvement in peer review, modifying review criteria | To adapt to a social landscape of greater accountability, dediscipline peer review by separating 'values' from 'facts', abolishing the notion that scientists can best assign social value to a certain health sphere, and include public representatives in decision-making | N/A (concept only) |  |
| Gallo et al (2013)[34] | Retrospective analysis | Biomedical sciences | USA | American Institute of Biological Sciences (AIBS), Scientific Peer Advisory and Review Services, decision-makers | virtual panels, teleconferencing | Reduce funder cost and time burden by replacing face-to-face funding panel discussions with teleconference panels | Trialling of teleconference funding committee meetings over a two-year period showed that they were as successful in supporting decision-making as face-to-face meetings, which led to them being implemented by the AIBS. | ** |
| Giraudeau et al (2011)[35] | Modelling | All research spheres | France | Broad funding infrastructure, decision-makers, | Modelling decision-making | Use the intraclass correlation coefficient formula to identify grant proposals with discordant reviewer ratings, which should require discussion prior to global scoring | N/A (concept only) |  |
| Gluckman (2012)[36] | Theoretical narrative | All research spheres | Australia, New Zealand | Broad funding infrastructure, HEIs, reviewers, applicants | Bibliometrics, modifying review criteria, improving applicant feedback, improving interrater reliability, reviewer selection, modifying panel composition, improving applicant feedback, reviewer accountability | Research team (and not investigator) track record is the best predictor of performance; proposals should be filtered for excellence first and relevance second; applicant feedback is more conducive to transparency than researcher training; small funding bodies must agree on their overarching priorities in grant allocation; the time reviewers take to assess proposals should be proportional to the quality of the proposal; interdisciplinarity requires increasing the panel size and expertise; reviewer justification should be minimised; interrater reliability requires independent reviewer assessment; focusing on individuals rather than projects helps build future research leaders. | N/A (concept only) |  |
| Gordon & Poulin (2009)[37] | Review | Hard sciences and engineering | Canada | National Science and Engineering Research Council, Discovery Grants Program, HEIs, applicants | Modifying funding allocation | Assume high scientific merit and accountability in all small proposals from tenured applicants: award all applicants base funds (e.g. $30,000), renew funding on a sliding scale or fund a mix of high-, medium- and low-risk grants | N/A (concept only) |  |
| Graves et al (2011)[38] | Retrospective analysis | Health and clinical research | Australia | NHMRC, HEIs, applicants | Modelling decision-making, | Formalise the element of chance in decision-making to increase transparency of and reduce bias within the process | N/A (concept only) |  |
| Gross & Bergstrom (2019)[39] | Modelling | All research spheres | USA | Broad research funding infrastructure, decision-makers, applicants | Random allocation of funding, bibliometrics | Award funds based on a lottery system, or the applicant's previous research success, to compensate for the 'loss of science value', time effort and financial burden of preparing proposals | N/A (concept only) |  |
| Gurwitz et al (2014)[40] | Theoretical narrative | Biomedical research | Israel, Austria & UK | Broad research funding infrastructure, reviewers | Reviewer and panel accountability, open access | Increase scientific transparency across the research community by publicising the identities of the panel members and reviewers, the reviews, the impact statements of the proposals and the final project reports | N/A (concept only) |  |
| Guthrie et al (2018)[41] | Review | All research fields | Global | Broad funding infrastructure, reviewers, decision-makers, programme officers | Golden ticket, lottery, modifying funding allocation, reviewer training in PRDM, virtual panels, teleconferencing, improving applicant feedback, shorter applications, using multimedia in proposals | Adopt some of the alternative approaches already implemented by research funders globally | Some globally implemented approaches: a reviewer training module on unconscious bias; online reviewer training to increase interrater reliability; gender equality in funding success; blinding of applicant identity and affiliations | ** (reported studies with long term outcomes) |
| Guthrie et al (2013)[42] | Review | All research fields | Global | Broad research funding infrastructure, applicants, programme leaders | Funder outreach to applicants, sandpits, modifying funding allocation, modelling decision-making virtual panels, random allocation of funds | Adopt some of the alternative approaches already implemented by research funders globally | The review concluded that at least three alternative funding strategies (funding renewals, milestone and portfolio funding) would still require a review process for effectiveness and efficiency. Funder mentoring of applicants helps strengthen unconventional proposals | ** (reported studies with long term outcomes) |
| Guthrie et al (2019)[43] | Review | All research fields | Global | Broad funding infrastructure, reviewers, programme officers, applicants | Reviewer training, improving interrater reliability, reviewer blinding, shorter applications, promoting innovation | Adopt some of the globally implemented alternative approaches relating to the evaluation and improvement of grant funding processes with regards to bias, burden and conservatism | While efforts to systematically measure the bias, burden and conservatism of peer review have overall been inadequate, there is evidence that: reviewer training on unconscious bias led to equal success in male and female experts; online training increased interrater reliability in new and experienced reviewers; and submitting shorter (two-page), anonymised proposals led to different funding decisions (see Bhattacharjee, 2012) | * |
| Hall et al (1992)[44] | Observational study and modelling | Health research | USA | NIH (National Cancer Institute), American Stop Smoking Intervention Study (ASSIST), federal and private research funders, public health research groups, HEIs, decision-makers, reviewers | Delphi, modelling decision-making, modifying review criteria | Use a formalised Delphi-based decision tool, comprising abstract primary and secondary criteria, to balance the long-term viability of an initiative with funding of many competing proposals and avoid political pressure and technical complexity regarding selecting a site for a study | Decision-makers for ASSIST chose one of the solutions proposed by the model and the approach was considered for extension to a larger NIH study. Long-term, ASSIST outcomes reported in 2003 showed a significant decrease in adult smoking - which, if implemented across all states, would have led a 1/4 million reduction in smokers nation-wide |  |
| Hansson & Monsted (2012)[45] | Review | All research spheres | Europe | European Commission, European Institute of Innovation and Technology, decision-makers | Modelling decision-making, simplifying PRDM, modifying review criteria | Use a Likert-based scaling decision model, instead of expert analysis of scientific merit, to simplify the review process | The authors concluded that the solution would hamper breakthroughs in science by reducing transparency, introducing politics into decision-making and removing scientific merit as a criterion | * |
| Herbert et al (2015)[46] | Prospective study | Health and medical research | Australia | NHMRC, Public Health and Basic Science Programmes, reviewers, decision-makers | Simplifying PRDM, modifying scoring of proposals | Simplify the review process (using three scores based on scientific quality, significance and innovation, and track record) to save time and cost that can then be reallocated to actual research | The simplified review process showed 75% agreement of funding outcomes with the traditional system, yet significantly reduced cost and time burden for reviewers and panellists | * |
| Holliday & Robotin (2010)[47] | Pilot | Health and clinical research | Australia | Cancer Council New South Wales, reviewers, decision-makers, applicants | Delphi, promoting innovation, modifying review criteria | Use a modified Delphi three-round scoring system (of scientific merit, innovation and risk) to prioritise funding of novel research and reduce political and social friction in decision-making | The Delphi process was successful as an efficient, transparent and equitable alternative method of review and was implemented for the New South Wales Pancreatic Cancer Network Strategic Research Partnership Grant | ** |
| Horlesberger et al (2013)[48] | Modelling and bibliometrics | Hard sciences and biomedical research | Europe | European Research Council, reviewers | Bibliometrics, promoting innovation | Use a model of quantitatively evaluating proposals, based on four-five ‘scientometric indicators of 'frontier research', to support the traditional review process | Adding the model to the review process would incur significant additional cost and time burden to the funder and reviewers. The model is also not able to extract the timeliness, risk and ‘pasteuresqueness’ factors of frontier research proposals | * |
| Huutoniemi (2012)[49] | Observational study | Social sciences and other spheres | Finland | Academy of Finland (AoF, a public funding body), reviewers, international experts, interdisciplinary applicants | Reviewer selection, improving interrater reliability, promoting interdisciplinary research | Interview and convene a multidisciplinary review panel of international experts in subject themes to promote interrater reliability and consensus (through collective scoring), and funding of interdisciplinary research | The AoF multidisciplinary panel funded every fourth proposal out of 109. Individual panellist opinions were validated by the group, which increased interrater reliability | * |
| Ismail et al (2009)[50] | Retrospective analysis | Biomedical research | UK, Europe | Medical Research Council, Department of Health and Social Care (Health Research & Development Policy Directorate), RAND (“Research ANd Development”) Europe, HEIs, national research charities, researchers, patients | Introducing submission quotas, limiting advertising for proposals, modifying funding allocation, promoting innovation, reviewer blinding, reviewer training in PRDM, reviewer accountability, open access | Promote funding of innovative and translational research using the US Defense Advanced Research Projects Agency (DARPA) and Canadian Health Services Research Foundation (CHSRF) models. Apply minor modifications to the funding process to reduce funding demand and improve the fairness, reliability and accountability of reviews | N/A (concept only) |  |
| Jaen and James (2008)[51] | Theoretical narrative | Health and clinical research | USA | Association of Departments of Family Medicine, NIH (Clinical and Translational Science), HEI faculty, clinicians, the public | Modifying panel composition, promoting community engagement, reviewer training in PRDM | Formally train and include faculty staff and clinicians in family medicine in the review process; allow more time for these stakeholders to review max. 10 proposals and attend panels, while relieving them of clinical and faculty duties. | N/A (concept only) |  |
| Jayasinghe et al (2001)[52] | Retrospective analysis | All research spheres | Australia | Australian Research Council | Reviewer selection, reviewer proposal matching | Authors recommended that a) researcher-nominated reviewers should not be selected, b) there should be more reviewers per proposal and c) a small selection of exceptional reviewers should perform the majority of reviews within a research theme to reduce error rates | Researcher-nominated external reviewers were biased and provided unreliable proposal ratings, although the ARC did not demonstrate any intrinsic bias in their review process | * |
| Johnston et al (2008)[53] | Theoretical narrative | Biomedical research | USA | NIH, reviewers, applicants | Shorter applications, limiting resubmissions, simplifying PRDM, continuous improvement of funding processes | Reduce application length, remove second resubmission attempts and reduce review length to increase reviewer numbers per proposal. Establish a rigorous research and continuous improvement programme for peer review. | N/A (concept only) |  |
| Juznic et al (2010)[54] | Modelling and bibliometrics | Hard science and engineering, health and biomedical research, social sciences and other spheres | Slovenia | The Slovenian Research Agency, Slovenian Current Research Information System, reviewers, decision-makers | Bibliometrics, reviewer accountability | Control for conflicts of interest during the review process and use biblio-/scientometric data to make funding decisions | Bibliometrics are used in the Slovenian funding landscape; however, they are less applicable to social sciences and humanities, or publications not written in English. Although the number of publications supports funding, it increases conflicts of interest at review - which should be avoided. | * |
| Kaiser (2003)[55] | Analysis of funder recommendation | Health and biomedical research | USA | NIH (Center for Scientific Review), High-Risk High-Reward Research Program, HEIs, applicants | Promoting innovation, modifying funding allocation, bibliometrics | Increase funding of unconventional research by exceptional researchers | The NIH set out a Congress-approved initiative to introduce the small Innovator Award to fund high-impact, innovative research by applicants who are leaders in their fields | * |
| Kaiser (2008)[56] | Pilot | Health and biomedical research | USA | NIH, HEIs, ECRs (new investigators) | Promoting new investigators, modifying funding allocation | Establish a quota of awards for new investigators to address the documented increasing average age at which researchers receive their first NIH award | In 2007, the pilot increased the funding success rate for new investigators to a level that was similar (~19%) to established applicants for the first time since 1995. This led to a new NIH target to equalise new grant success rates among the two applicant groups | * |
| Kaiser (2008)[57] | Analysis of funder recommendation | Health and biomedical research | USA | NIH, HEIs, reviewers, decision-makers, applicants | Shorter applications, modifying review criteria, improving applicant feedback, reviewer incentives | Reduce application size from 25 to 12 pages (shifting focus from methodological detail to impact) and transform the overall score into five criterion-based scores to improve feedback for applicants. Award reviewers extra funding as an incentive | N/A (concept only) |  |
| Kaiser (2008)[58] | Analysis of funder recommendation | Health and biomedical research | USA | NIH, HEIs, reviewers, decision-makers, applicants | Limiting resubmissions, introducing submission quotas, promoting new investigators | Mark applications that are not recommended for resubmission; consider amended applications as new; consider a separate review process for new investigator grants; limit the number of grants awarded per investigator | N/A(concept only) |  |
| Kaplan et al (2008)[59] | Modelling | Health and biomedical research | USA | NIH, HEIs, reviewers | Reviewer proposal matching, modelling decision-making, modifying scoring of proposals | Determine mathematically the number of reviewers required to accurately score grant applications, according to the NIH-standard three significant figures | The model showed that a large number of reviewers would be required to increase scoring accuracy, decide between applications that vary in score by <1, and accurately assess non-conventional (innovative) proposals | * |
| Kennedy (2008)[60] | Analysis of funder recommendation | Health and biomedical research | USA | NIH, HEIs, reviewers, applicants | Limiting resubmissions, shorter applications, reviewer accountability, continuous improvement of funding processes | Remove the 'special status' of amended applications by considering them as new at review. Shorten the application summary statements to focus on the scientific merit only, minimising the application length | The NIH recommended to: reduce administrative burden on applicants, reviewers and NIH staff; enhance the rating system; enhance review and reviewer quality; optimise support for different career stages/types; optimise support for different types/approaches of science; reduce the stress on the support system of science; and re-evaluate peer review. | * |
| Kight (2010)[61] | Observational study and modelling | All research spheres | Global | Broad funding infrastructure, decision-makers, programme officers, applicants | Modelling decision-making, panel accountability, modifying review criteria | Employ a quantitative model for predicting the 'reasonableness' (or feasibility) of a proposal to support, standardise and reduce the bias of decision-making | The PhD thesis concludes that research scope, resources, cost and schedule are predictors of the 'reasonableness' of a proposal. The expected social impact of this work is to improve insight for applicants, more efficiently allocate funding for and prioritise poorly funded research areas (e.g. social sciences) and further study models of research feasibility | * |
| Kobayashi (2000)[62] | Retrospective analysis | All research spheres | Japan | Broad funding infrastructure (private, government and public research sectors), HEIs, applicants, decision-makers | Modifying review criteria, promote sponsoring of research | Adapt the 'audition system' from the Performing Arts to Research & Development to unite sponsors of social priorities and the research community across the academic, public and private research sectors. | There is evidence that this audition system has been adopted by countries with recently introduced technologies and R&D in areas such as defence and health - where market mechanisms are insufficient. | ** |
| Kroto (2010)[63] | Theoretical narrative | All research spheres | Global | Broad research funding infrastructure, applicants, ECRs | Promoting ECRs, modifying funding allocation, transfer funding decisions to HEIs | Eliminate the grant proposal and review system; instead, let local academic departments split funding as they best see fit among ECRs (who represent the future), investigators with a recent record of research excellency (the 'racehorse' approach) and those who have not been funded but are in a proposal with the second group. | N/A (concept only) |  |
| Krumholz (2013)[64] | Theoretical narrative | Health and clinical research | USA | Yale University School of Medicine, Yale School of Public Health, Yale-New Haven Hospital, Robert Wood Johnson Foundation, Clinical Scholars Program, Center for Outcomes Research and Evaluation, HEIs, applicants, reviewers | Modifying review criteria, increasing the application length | Introduce methodological appendices in proposals for reviewers to make a determination, based on the technical details, of the potential replicability and true value of the research. This would help eliminate low impact projects that wouldn't be published within three years. | N/A (concept only) |  |
| Kupfer et al (2014)[65] | Retrospective analysis | Health and clinical research | USA | NIH, Department of Psychiatry at the University of Pittsburgh, HEIs, applicants, reviewers | Introducing submission quotas, promoting collaboration, promoting interdisciplinary research, improving applicant feedback | Institutions should adopt an internal systematic peer review system for proposals prior to their submission to the NIH | The University's internal Research Review Committee successfully implemented internal peer review in 2011. All academics participated as both reviewers and applicants; the effect of this was an increase in intra- and interdepartmental collaborations and publications arising from the collective sharing of research strategies. | ** |
| Lane (2010)[66] | Observational study | All research spheres | USA | NSF, NIH, broad funding landscape, decision-makers, applicants | Bibliometrics, open access | Funders should provide applicants with a standardised template for reporting scientific achievements to improve data quality. Data collected for bibliometric analysis should be openly accessible by the research community for reproducibility; funders should achieve broader research access to data from publishers, compensating them for their citation documentation costs. | A pilot of the NSF- and NIH-funded STAR METRICS (Science and Technology in America’s Reinvestment - Measuring the Effects of Research on Innovation, Competitiveness and Science) project, conducted at six institutions, showed that automation could substantially cut data collection times for investigators. | ** |
| Laudel (2006)[67] | Observational study and modelling | All research spheres | Germany | Sonderforschungsbereiche (SFB; collaborative research network), Deutsche Forschungsgemeinschaft (DFG; national research funder), HEIs, private research sector, applicants | Promoting interdisciplinary research, promoting collaboration | Create a research network between funders, reviewers and applicants to promote funding of multidisciplinary and collaborative research. | None of the researchers within the SFB network (even those whose proposals were rejected for funding) expressed any concern regarding the competency of reviewers in assessing multidisciplinary proposals | * |
| Lenard (2006)[68] | Theoretical narrative | Health and biomedical research | USA | NIH (Study Sections), HEIs, applicants (senior faculty), decision-makers | Modifying panel composition | All NIH grant holders above the rank of assistant professor must participate in the NIH funding committee evaluations – as a formal condition to receive NIH funding. | N/A (concept only) |  |
| Mayo et al (2006)[69] | Pilot | Health and clinical research | USA | McGill University Health Centre, HEIs, medical organisations, ECRs, applicants | Improving interrater reliability, modifying scoring of proposals, panel accountability | Replace the traditional approach to application ranking by two main reviewers with a system where all reviewers rank a proposal and then apply a consensus score to make decisions. | Comparison of the classic structured scientific in-depth two-reviewer critique (CLASSIC) with the all-panel-member independent ranking method (RANKING) showed that the former was associated with a considerable degree of chance in funding, while the latter abolishes the impact of outlier reviews and should therefore be the preferred method | * |
| Mervis (2014)[70] | Pilot | Hard sciences and engineering | USA | NSF (Civil, Mechanical and Manufacturing Innovation Division, CMMID), programme officers, applicants, reviewers | Reviewer selection, reviewer incentives, improving interrater reliability, modifying scoring of proposals | Each grant applicant should review seven proposals from peers who are competing for the same grant and rank them from best to worst. Honest reviews and interrater consensus will score applicants points on their own proposals. | The pilot produced a highly motivated pool of reviewers, which saved time and costs for the NSF's CMMID program managers and maintained a review quality that was within NSF standards for traditional peer review. This led the NSF to consider expanding the pilot to its other programs. | * |
| Navascues & Budroni (2019)[71] | Modelling | Hard sciences | Belgium | Broad funding infrastructure, applicants, decision-makers | Modelling decision-making, bibliometrics | Develop a mathematical model of 'scientific productivity', based on the applicant’s recent research activity, to facilitate decision-making in the funding of theoretical sciences | N/A (concept only) |  |
| Obrecht et al (2007)[72] | Observational study | Health and biomedical research | Canada | Canadian Institutes of Health Research, Standing Committee, decision-makers | Remote panels | Determine whether the decision value of panel discussions is worth the time burden for decision-makers by assigning them at-home evaluations of the proposals prior to the panel | Face-to-face panel discussion and scoring of proposals did not significantly improve the fairness and effectiveness of funding decisions post-review, and the presence of panellists who have not read the proposals at hand was found to pose the greatest threat to the fairness of decisions | * |
| Olbrecht & Bornmann (2010)[73] | Review | Social science and other spheres | USA, Canada, Norway, Germany | Broad funding infrastructure, HEIs, decision-makers | Panel accountability, promoting interdisciplinary research, reviewer proposal matching | Minimise the psychological factors hindering group decisions by: declaring panellist expertise; providing unshared information to the panel; assigning each reviewer responsibility for different parts of a proposal; moderating discussions into information search, integration and decision components; ensuring sufficient input from all members; assigning a member to play 'devil's advocate'; ensuring group diversity | The three measures of mitigating negative group effects in panel review pertaining to 1) organisation of a group meeting, 2) moderation of group discussion and 3) the composition of the group were found to be effective in the case studies. The authors suggested research questions for further empirical studies into social psychology in peer review | * |
| Pabersz et al (2014)[74] | Pilot | Health and clinical research | USA | Michigan Institute for Clinical and Health Research, Community-University Research Partnerships, Community Engagement Programme (CEP), Community-Engaged Research, Clinical and Translational Science Award (CTSA), HEIs, reviewers, applicants, the public (community health) | Patient and public involvement in PRDM, promoting clinical research | Improve the quality of clinical research by integrating the community's knowledge into peer review; include 10-12 community partners and 4-6 HEI staff in review panels across multiple rounds of clinical research funding | The five-year pilot was directly responsible for improving the CEP, with recommendations made to further strengthen the review process in clinical research based on community and HEI partner insight. Survey with CEP staff revealed that 88% of members requested better definitions of the review criteria | ** |
| Petsko (2006)[75] | Theoretical narrative | Biomedical sciences | USA | NIH, HEIs, applicants, reviewers, programme officers, decision-makers | Reviewer selection, shorter applications, interdisciplinary reviewers, reviewer accountability, autonomy in decision-making | Require grant-holders to serve on review panels; require new, but not established, investigators to detail methodology in their proposals; discourage review in the same field of expertise, except for technical evaluation; review the quality of reviews; allow programme officers more autonomy in decision-making; and reduce the proposal size | N/A (concept only) |  |
| Pina et al (2015)[76] | Retrospective analysis | Hard sciences, social sciences and other spheres | Europe | Marie Sklodowska-Curie Actions, EU Seventh Framework Programme for Research, European Commission, HEIs, reviewers, decision-makers | Improving interrater reliability, remote panels, modifying scoring of proposals | Apply a two-tiered process of proposal evaluation, including an individual evaluation report (IER), carried out remotely by reviewers, and a consensus report (CR) carried out in person by the same reviewers. | Between 2007 and 2013, evaluation of 24,897 proposals showed high correlation between IER and CR scores across different grant schemes and panels. Disagreement between scores was more frequent in the social sciences | ** |
| Pollitt et al (1996)[77] | Pilot | Health research | USA | NIH (National Institute of Mental Health), reviewers | Introduce classification of criticisms in peer review | Employ a classification system of criticisms received following review of rural mental health research grants (as this research may be more difficult to conduct in rural, rather than urban, areas) | Review of 24 grant application generated 557 criticisms in the summary statements, distributed as follows: inadequate description of measurements (7%); inadequate justification for study (5%); inadequate justification for chosen analyses (4%); and inappropriate research design (4%). There was insufficient evidence, however, that rural research requires special review standards | * |
| Potvin (2019)[78] | Retrospective analysis | Other sphere (digital humanities) | USA | University of West Virginia, digital humanities (DH) research and science communication, librarians, editors, reviewers, funders | Reviewer selection, reviewer accountability, open access, improving applicant feedback | Promote multidisciplinary reviewer selection, interrater transparency and open exchange using pre-print archives (such as arXiv, bioRxiv), mega-journals (such as PeerJ) and open libraries. Restructure peer review from a top-down to cross-community hierarchy at the conference level by widening access to proposals | The DH conference’s reform in 2012 allowed wider reviewer access to comments on proposals and gave reviewers the option to choose which reviews to review. This promoted evaluation and exchange and has led to a formalised feedback system for authors looking to appeal their reviews | ** |
| Pupella et al (2014)[79] | Feasibility study | Health research | Italy | Italian Cancer Network, Training through Research Application Italian iNitiative (TRAIN), European Commission Seventh Framework Program, HEIs, applicants, reviewers, editors | Reviewer selection, reviewer matching to proposals, bibliometrics, automating funder processes | Employ a web-based semi-automatic tool for reviewer selection that uses the citation index of reviewers and matches MeSH descriptors from reviewer publications to the descriptors linked to the keywords in proposals. Require fellowship applications to include a PubMed ID and 20 most relevant publications | The web-based selection tool led to 162 reviewers being discarded, and a further 23 excluded for insufficient citation indices, resulting in a total register of 205 reviewers. Only 45 reviewers (22%) demonstrated an unwillingness to participate in the study by refusing to upload publications | * |
| RAND (2012)[80] | Review | Biomedical research | UK, Europe | RAND Europe, Policy Research in Science and Medicine (PRiSM), Natural Environment Research Council (NERC), Engineering and Physical Sciences Research Council (EPSRC), broad funding landscape, reviewers, programme officers | see Ismail et al (2009) | see Ismail et al (2009) | The Research Council of Norway now has a single annual deadline and the Wellcome Trust pre-selects eligible institutions for its Prize Studentships, also limiting nominations per institution. NERC limits applications for studentships and the EPSRC also employs eligibility criteria to limit demand. There is a lack of evidence on how extensively the Ismail et al (2009) modifications have contributed to improving peer review, and any effect on funding demand has deemed marginal by RAND. | ** |
| Research Councils UK (RCUK, 2006) [81] | Retrospective analysis, observational study and modelling | All research spheres | UK | RCUK (now UK Research Institute), Research Councils (8 in total), Department of Business, Energy and Industrial Strategy (BEIS), HEIs, applicants, reviewers | Introducing submission quotas, modifying funding allocation, limiting resubmissions | Cut costs by increasing the size of RCUK awards (to 3-5 years), introducing institutional-level quotas for all or poor-performing HEIs (and distributing success rates), limiting resubmissions and introducing a research outline bid stage prior to full proposal submission to the review process | N/A (concept only) |  |
| Research Councils UK (2007) [82] | Observational study | All research spheres | UK | University and College Union (UCU), HEI faculty (managers, librarians, administrative and IT staff), wider research community | Promoting career stability, modifying funding allocation, promoting ECRs, modifying review criteria, reviewer training in PRDM, reviewer selection | A counter-proposal to the RCUK (2006) report: introduce permanent/open-ended contracts for researchers; retain small grants in low-budget disciplines; prevent further 'concentration' of funding at top 10% institutions by basing awards on application quality and not affiliations; increase reviewer pool to promote multidisciplinary research funding; introduce virtual training in review |  | */** |
| Research Councils UK (2007)[83] | Retrospective analysis | All research spheres | UK | RCUK, BEIS, UCU, Societies, Russel Group and other UK HEIs, research community stakeholders | See RCUK (2007) above + promoting innovation, maximising information use | Modifying funding allocation (increasing grant duration and size proportionately to track record), modifying research group size; promoting ECR and innovation funding, limiting resubmission (for high-merit proposals), shorter applications (in the arts, humanities and social sciences); applicant training, maximising information use (from final reports to inform future applications) | In response to the community-wide stakeholder consultation on the RCUK report (RCUK, 2006), RCUK modified recommendations for funding and peer review reforms. There is evidence that other trusts employ the interventions proposed by the RCUK and UCU (e.g. use of short proposals by the Leverhulme Trust, Wellcome Trust, NERC and AHRC) | ** |
| Roebber (2011)[84] | Modelling | All research spheres | USA, UK and Finland | Broad funding infrastructure, HEIs, decision-makers | Modelling decision-making, introducing submission quotas, reviewer selection | Employ an agent-based simulated funding model for testing the efficiency of different funding strategies by accounting for both reviewer and funder behaviour | The simulation highlighted the aspects of the funding process that most determine its efficacy: the program officer's approach and the funder policy. Funding demand can be reduced by increasing the reviewer pool and introducing a cooling-off period, or application quota, for investigators. | * |
| Ronai (2012)[85] | Theoretical narrative | Health and biomedical research | USA | NIH, broad funding infrastructure, HEIs, applicants, reviewers, decision-makers | Bibliometrics, modifying review criteria | Change how the applicant track record is used to assess the likelihood of research success: rather than the quantity, assess the quality of publications (e.g. insightfulness, novel technology, paradigm shifts) and identify any gaps in track record that may highlight research issues | N/A (concept only) |  |
| Russell et al (1983)[86] | Pilot | Health and biomedical research | Canada | Canadian health research, Arthritis Society, decision-makers, reviewers, applicants | Simplifying PRDM, shorter applications, improving interrater reliability | Replace detailed review of full proposals with simplified (preliminary) review of single-page summaries including applicant affiliations | There was a strong correlation between scores following preliminary and detailed review of proposals, showing that detailed discussions of full proposals was not necessary for decision-making | * |
| Schroter et al (2004)[87] | Trial | Medical research | UK | British Medical Journal, NHS London Regional Office Research and Development Directorate, editors, reviewers | Reviewer training | Improve ex-post review quality by training reviewers on what editors want from a review using taught workshops or self-taught training packages | Overall, training improved review quality: both groups identified more errors in proposals and recommended more manuscripts for rejection compared to controls. However, short training appears to be insufficient to significantly improve review quality and a larger trial of longer interventions is therefore warranted | * |
| Schroter et al (2010)[88] | Observational study | Biomedical research | Global (19 countries) | International public and private biomedical research funders (n=57; 49% participation rate), reviewers (62% participation rate), programme officers | Standardising peer review, reviewer training in PRDM, reviewer incentives | Use globally collected feedback from research funders and external reviewers to identify key areas where the review process can be improved and standardised | 60% of funders agreed that standardising peer review would be of value. 85% of reviewers reported a lack of training in peer review and 64% expressed a desire to a have access to formal training. Reviewers also highlighted the need to reduce the time burden of review, provide academic recognition to reviewers and increase the level of support from funders | * |
| Sinkjaer (2018)[89] | Retrospective analysis | All research spheres | Denmark | Danish National Research Foundation, the Villum Foundation, ECRs, reviewers, programme officers | Promoting innovation, shorter applications, Golden ticket, reviewer blinding | Support innovation by shortening the proposal to a three-page summary, blinding reviewers to the applicant's track record and allocate each reviewer 'a Golden ticket' to fund a proposal of their choice | The 2017 funding scheme at VF led to a 10% funding rate (39 grants for up to 2 years), where a third of grants were awarded to investigators under 40 years of age and 31% of proposals were funded as golden tickets. Half of those Golden ticket proposals would not have been funded otherwise. Reviewer and applicant feedback reflected positively on proposal anonymity. | * |
| Shepherd et al (2018)[90] | Review | Health and biomedical research | UK, Australia, Canada and USA | NIHR and global funders (including AusHSI and NHMRC), applicants, reviewers, programme officers | Shorter grant proposals, simplified scoring, accelerated funding process, streamlined review process | Shorten proposals, use smaller reviewer panels and expedite reviews and funding decisions to reduce administrative costs | The systematic map of innovations in peer review was disseminated to the NIHR Working Group, comprising 11 members across all NIHR centres |  |
| Smith et al (2013)[91] | Pilot | Health research | USA | NIH, University of Wisconsin Institute for Clinical and Translational Research, HEIs, reviewers, the public | Patient and public involvement in PRDM, promoting community engagement, applicant training in PRDM | Address the gap in community health engagement and type-2 translational research (practice-and community-based) by introducing an external community review committee to re-rank scientifically reviewed proposals prior to decision-making | The pilot award scheme successfully funded 41 applications, leading to a $20M return in funds and 16 publications). 21% of the funded proposals were from ECRs, who required 4 months of training prior to submission. Community reviewers were comfortable re-rating scientifically reviewed proposals without prior indication of merit and conflicts were effectively settled via open communication | * |
| Suls and Martin (2009)[92] | Theoretical narrative | All research spheres | USA | Broad research infrastructure | Reviewer accountability, open access | Eliminate the risk of malice, subjectivity and errors in review by introducing an open review policy, as well as rapid access to and open exchange of pre-prints between reviewers (with moderation from editors), as in the physics and artificial intelligence fields | N/A (concept only) |  |
| Thornley et al (2002)[93] | Pilot | Health and clinical research | Canada | Alberta Heritage Foundation for Medical Research (AHFMR), HEIs, ECRs and students | Modelling decision-making, modifying review criteria | Integrate the ProGrid decision-assist software into funding of trainee grants, which incorporates the following criteria into decision-making: academic record, research experience, reference letters, supervisor background and resources, role of the trainee, the overall merit of the project and the training environment | The initiative led to good applicant feedback, simplified decision-making and a reduction in administrative burden and committee meeting lengths. Use of the ProGrid tool was then extended to other trainee programs within the AHFMR | ** |
| Whaley (2006)[94] | Pilot | Mental health research | USA | Hogg Foundation at University of Texas at Austin, broad funding infrastructure, HEIs, reviewers | Improving interrater reliability, modifying scoring of proposals | Determine the ecological validity of the established and psychometrically valid Grant Proposal Rating Form (GPRF), which consists of multiple items, to implement consistent and objective guidelines for peer review | Total scores are more reliable for assessing proposals than subscales, regardless of the proposal length; however, the reliability of scoring large items (such as mental health) is inconsistent. Although the adequacy of the research plan was consistently rated across subscales, (making it a reliable item), factors other than the applicant's research plan influenced rating - such as applicant experience in shorter grant applications with less scientific detail. | * |
| Wooding & Guthrie (2017)[95] | Analysis of funder implementation | Biomedical research | Europe, Canada, UK | Canadian Institutes of Health Research (CIHR), RAND, Health Canada, University of Cambridge, programme officers, reviewers, applicants | Virtual panels, introducing submission quotas, improving applicant feedback, panel rotation, panel accountability | Employ an international review panel and an external consultation to examine the empirical evidence on the best approaches to tackling burdens in grant funding. Reduce funder and reviewer burden by introducing virtual discussion forums | Issues with restructuring the CIHR scheme and reduced overall funding led to a community-wide protest letter to the Canadian Health Minister. The international review panel and RAND recommended the following solutions: a proposal triage stage; improving applicant feedback; better training for funder administrative staff; a rotating panel of more international members; continuing to test the value of virtual decision panels | ** |

**Publication included after peer review (n=1)**

| **Author(s) & year** | **Study design** | ***Contexts*** | | | ***Mechanisms*** | | ***Outcomes*** | ***Short-term****  ***Long-term** outcome(s)*** |
| --- | --- | --- | --- | --- | --- | --- | --- | --- |
|  |  | **Research field** | **Location** | **Stakeholders involved** | **Intervention type** | **Intervention focus** |  |  |
| Sattler et al (2015)[96] | Randomised controlled trial evaluation brief training programme for reviewers of NIH research grant applications | Public Health research | USA | Seventy-five Public Health professors from U.S. research universities | Experimental. Peer review training  on the NIH rating scale and research grant proposal scoring system | Increasing knowledge of rating scale, inter-rater reliability of reviewers’ scores, and time spent reading review criteria | The training programme increased participants’ scoring accuracy, inter-rater reliability, and the amount of time reading the review criteria compared to the participants in the no training condition. | * |

References

1. Abramo, G., C.A. D'Angelo, and F. Viel, *Selecting competent referees to assess research projects proposals: A study of referees' registers.* Research Evaluation, 2013. **22**(1): p. 41-51.

2. Ahmed, S.M. and A.G. Palermo, *Community engagement in research: frameworks for education and peer review.* American Journal of Public Health, 2010. **100**(8): p. 1380-7.

3. Albanese, M., et al., *An institutionally funded program for educational research and development grants: it makes dollars and sense.* Academic Medicine, 1998. **73**(7): p. 756-61.

4. AMRC (Association of Medical Research Charities), *A house in good order: a report on the AMRC peer review audit 2011*. 2012: London, UK.

5. Andejeski, Y., et al., *Quantitative impact of including consumers in the scientific review of breast cancer research proposals.* Journal of Womens Health & Gender-Based Medicine, 2002. **11**(4): p. 379-88.

6. Avin, S., *Why we still need grant peer review.* EMBO Reports, 2014. **15**(5): p. 465-6.

7. Avin, S., *Mavericks and lotteries.* Studies in History and Philosophy of Science Part A, 2019. **76**: p. 13-23.

8. Barnett, A.G., et al., *Streamlined research funding using short proposals and accelerated peer review: an observational study.* BMC Health Services Research, 2015. **15**: p. 55.

9. Bhattacharjee, Y., *Science funding. NSF's 'Big Pitch' tests anonymized grant reviews.* Science, 2012. **336**(6084): p. 969-70.

10. Bielski, A., R. Harris, and N. Gillis, *Summary report of comments received on NIH system to support biomedical and behavioral research and peer review*. 2007: Bethesda, MD, USA.

11. Bollen, J., et al., *From funding agencies to scientific agency: Collective allocation of science funding as an alternative to peer review.* EMBO reports, 2014. **15**(2): p. 131-133.

12. Bonetta, L., *Growing pains for NIH grant review.* Cell, 2006. **125**(5): p. 823-5.

13. Bonetta, L., *Enhancing NIH grant peer review: a broader perspective.* Cell, 2008. **135**(2): p. 201-4.

14. Boyack, K.W., M.C. Chen, and G. Chacko, *Characterization of the peer review network at the Center for Scientific Review, National Institutes of Health.* PLoS ONE [Electronic Resource], 2014. **9**(8): p. e104244.

15. Brumfiel, G., *Rethink on review leaves researchers out in the cold.* Nature, 2005. **435**(7038): p. 5.

16. Cabezas-Clavijo, A., et al., *Reviewers' ratings and bibliometric indicators: hand in hand when assessing over research proposals?* PLoS ONE [Electronic Resource], 2013. **8**(6): p. e68258.

17. Canibano, C., J. Otamendi, and I. Andujar, *An assessment of selection processes among candidates for public research grants: the case of the Ramon y Cajal Programme in Spain.* Research Evaluation, 2009. **18**(2): p. 153-161.

18. Carpenter, A.S., et al., *A retrospective analysis of the effect of discussion in teleconference and face-to-face scientific peer-review panels.* BMJ Open, 2015. **5**(9): p. e009138.

19. Cechlarova, K., T. Fleiner, and E. Potpinkova, *Assigning evaluators to research grant applications: the case of Slovak Research and Development Agency.* Scientometrics, 2014. **99**(2): p. 495-506.

20. Chen, Y.-C. and C.-C. Tsai, *An educational research course facilitated by online peer assessment.* Innovations in Education and Teaching International, 2009. **46**(1): p. 105-117.

21. Cherfas, J., *Peer Review: Software for Hard Choices: Some agencies in the U.K. are experimenting with an electronic system that helps make funding decisions.* Science, 1990. **250**(4979): p. 367-8.

22. Clarke, P., et al., *A randomized trial of fellowships for early career researchers finds a high reliability in funding decisions.* Journal of Clinical Epidemiology, 2016. **69**: p. 147-51.

23. De Los Reyes, A. and M. Wang, *Applying psychometric theory and research to developing a continuously distributed approach to making research funding decisions.* Review of General Psychology, 2012. **16**(3): p. 298-304.

24. Demicheli, V. and C. Di Pietrantonj, *Peer review for improving the quality of grant applications.* Cochrane Database of Systematic Reviews, 2007(2): p. MR000003.

25. Dumanis, S.B., et al., *It's money! Real-world grant experience through a student-run, peer-reviewed program.* CBE Life Sciences Education [Electronic Resource], 2013. **12**(3): p. 419-28.

26. Durso, T.W., *Researchers disagree on NIH plan to improve its peer-review process.* Scientist, 1996. **10**(24): p. 1-&.

27. Fang, F.C. and A. Casadevall, *Research Funding: the Case for a Modified Lottery.* mBio, 2016. **7**(2): p. e00422-16.

28. Fielder, A. and H. Vinyard, *Peer review of grant applications.* Lancet, 1998. **352**(9133): p. 1063.

29. Fleurence, R.L., et al., *Engaging patients and stakeholders in research proposal review: the patient-centered outcomes research institute.* Annals of Internal Medicine, 2014. **161**(2): p. 122-30.

30. Forsdyke, D.R., *Bicameral grant review: an alternative to conventional peer review.* FASEB Journal, 1991. **5**(9): p. 2313-4.

31. Foundation), N.N.S., *Proposal management efficiencies*. 2017.

32. Freireich, E.J., *A study of the status of clinical cancer research in the United States (1990).* Journal of the National Cancer Institute, 1991. **83**(12): p. 829-37.

33. Frodeman, R. and A. Briggle, *The Dedisciplining of Peer Review.* Minerva, 2012. **50**(1): p. 3-19.

34. Gallo, S.A., A.S. Carpenter, and S.R. Glisson, *Teleconference versus face-to-face scientific peer review of grant application: effects on review outcomes.* PLoS ONE 2013. **8**(8): p. e71693.

35. Giraudeau, B., et al., *Peer review of grant applications: a simple method to identify proposals with discordant reviews.* PLoS ONE [Electronic Resource], 2011. **6**(11): p. e27557.

36. Gluckman, P., *Which science to fund: time to review peer review?* 2012.

37. Gordon, R. and B.J. Poulin, *Cost of the NSERC Science Grant Peer Review System exceeds the cost of giving every qualified researcher a baseline grant.* Accountability in Research, 2009. **16**(1): p. 13-40.

38. Graves, N., A.G. Barnett, and P. Clarke, *Funding grant proposals for scientific research: retrospective analysis of scores by members of grant review panel.* BMJ, 2011. **343**: p. d4797.

39. Gross, K. and C.T. Bergstrom, *Contest models highlight inherent inefficiencies of scientific funding competitions.* PLoS biology, 2019. **17**(1): p. e3000065.

40. Gurwitz, D., E. Milanesi, and T. Koenig, *Grant application review: the case of transparency.* Plos Biology, 2014. **12**(12): p. e1002010.

41. Guthrie, S., I. Ghiga, and S. Wooding, *What do we know about grant peer review in the health sciences? [version 2; peer review: 2 approved].* F1000Research, 2018. **6**(1335).

42. Guthrie, S., et al., *Alternatives to peer review in research project funding*. 2013: Cambridge, UK.

43. Guthrie, S., et al., *Measuring bias, burden and conservatism in research funding processes [version 1; peer review: awaiting peer review].* F1000Research, 2019. **8**(851).

44. Hall, N.G., et al., *A model for making project funding decisions at the National Cancer Institute.* Operations Research, 1992. **40**(6): p. 1040-52.

45. Hansson, F. and M. Monsted, *Changing the Peer Review or Changing the Peers - Recent Development in Assessment of Large Research Collaborations.* Higher Education Policy, 2012. **25**(3): p. 361-379.

46. Herbert, D.L., et al., *Using simplified peer review processes to fund research: a prospective study.* BMJ Open, 2015. **5**(7): p. e008380.

47. Holliday, C. and M. Robotin, *The Delphi process: a solution for reviewing novel grant applications.* International journal of general medicine, 2010. **3**: p. 225-30.

48. Horlesberger, M., et al., *A concept for inferring 'frontier research' in grant proposals.* Scientometrics, 2013. **97**(2): p. 129-148.

49. Huutoniemi, K., *Communicating and compromising on disciplinary expertise in the peer review of research proposals.* Social Studies of Science, 2012. **42**(6): p. 897-921.

50. Ismail, S., A. Farrands, and S. Wooding, *Evaluating grant peer review in the health sciences. A review of the literature*. 2009: Cambridge, UK.

51. Jaen, C.R., P. James, and M. Association of Departments of Family, *You have to be in to win: presenting family medicine's perspective in NIH scientific reviews.* Annals of Family Medicine, 2008. **6**(2): p. 179-80.

52. Jayasinghe, U.W., H.W. Marsh, and N. Bond, *Peer review in the funding of research in higher education: The Australian experience.* Educational Evaluation and Policy Analysis, 2001. **23**(4): p. 343-364.

53. Johnston, S.C. and S.L. Hauser, *Peer review at National Institutes of Health: Small steps forward.* Annals of Neurology, 2008. **64**(5): p. A15-A17.

54. Juznic, P., et al., *Scientometric indicators: peer-review, bibliometric methods and conflict of interests.* Scientometrics, 2010. **85**(2): p. 429-441.

55. Kaiser, J., *Biomedical research. NIH plans new grants for innovative minds.* Science, 2003. **301**(5635): p. 902.

56. Kaiser, J., *National Institutes of Health. Zerhouni's parting message: make room for young scientists.* Science, 2008. **322**(5903): p. 834-5.

57. Kaiser, J., *National Institutes of Health. Changes in peer review target young scientists, heavyweights.* Science, 2008. **320**(5882): p. 1404.

58. Kaiser, J., *Peer review. NIH urged to focus on new ideas, new applicants.* Science, 2008. **319**(5867): p. 1169.

59. Kaplan, D., N. Lacetera, and C. Kaplan, *Sample size and precision in NIH peer review.* PLoS ONE [Electronic Resource], 2008. **3**(7): p. e2761.

60. Kennedy, D.N., *New happenings at the NIH.* Neuroinformatics, 2008. **6**(2): p. 69-70.

61. Kight, W.D., *An analysis of reasonableness models for research assessments*. 2010, ProQuest Information & Learning: US. p. 1004-1004.

62. Kobayashi, S., *Applying audition systems from the performing arts to R&D funding mechanisms: quality control in collaboration among the academic, public, and private sectors in Japan.* Research Policy, 2000. **29**(2): p. 181-192.

63. Kroto, H.W., *Working at the coal face.* Nature, 2010. **467**(7317): p. S13.

64. Krumholz, H.M., *Grant applications with a result-based orientation.* Circulation. Cardiovascular Quality & Outcomes, 2013. **6**(5): p. 507-8.

65. Kupfer, D.J., et al., *Using peer review to improve research and promote collaboration.* Academic Psychiatry, 2014. **38**(1): p. 5-10.

66. Lane, J., *Let's make science metrics more scientific.* Nature, 2010. **464**(7288): p. 488.

67. Laudel, G., *Conclave in the Tower of Babel: how peers review interdisciplinary research proposals.* Research Evaluation, 2006. **15**(1): p. 57-68.

68. Lenard, J., *Two facets of peer review and the proper role of study sections.* Accountability in Research, 2006. **13**(3): p. 277-83.

69. Mayo, N.E., et al., *Peering at peer review revealed high degree of chance associated with funding of grant applications.* Journal of Clinical Epidemiology, 2006. **59**(8): p. 842-8.

70. Mervis, J., *Research grants. A radical change in peer review.* Science, 2014. **345**(6194): p. 248-9.

71. Navascués, M. and C. Budroni, *Theoretical research without projects.* PloS one, 2019. **14**(3): p. e0214026.

72. Obrecht, M., K. Tibelius, and G. D'Aloisio, *Examining the value added by committee discussion in the review of applications for research awards.* Research Evaluation, 2007. **16**(2): p. 79-91.

73. Olbrecht, M. and L. Bornmann, *Panel peer review of grant applications: what do we know from research in social psychology on judgment and decision-making in groups?* Research Evaluation, 2010. **19**(4): p. 293-304.

74. Paberzs, A., et al., *Strengthening community involvement in grant review: insights from the Community-University Research Partnership (CURES) pilot review process.* Clinical and translational science, 2014. **7**(2): p. 156-63.

75. Petsko, G.A., *Instructions for repair.* Genome Biology, 2006. **7**(4): p. 106.

76. Pina, D.G., D. Hren, and A. Marusic, *Peer Review Evaluation Process of Marie Curie Actions under EU's Seventh Framework Programme for Research.* PLoS ONE [Electronic Resource], 2015. **10**(6): p. e0130753.

77. Pollitt, F.A., C.M. Notgrass, and C. Windle, *Peer review of rural research grant applications.* Administration and Policy in Mental Health, 1996. **24**(2): p. 173-180.

78. Potvin, S., *And Who Will Review the Review(er)s?* College & Research Libraries, 2017. **78**(6).

79. Pupella, V., et al., *A semi-automatic web based tool for the selection of research projects reviewers.* Studies in Health Technology & Informatics, 2014. **205**: p. 950-4.

80. RAND Europe, *Evaluating grant peer review. Key findings of a literature review of grant peer review in the health sciences*. 2012: Cambridge, UK.

81. RCUK (Research Councils UK), *Report of the Research Councils UK Efficiency and Effectiveness of Peer Review project*. 2006: Swindon, UK.

82. RCUK (Research Councils UK), *RCUK Response to the project report & consultation on the efficiency and effectiveness of peer review*. 2007: Swindon, UK.

83. RCUK (Research Councils UK), *Summary of the analysis of the responses received to the RCUK efficiency and effectiveness of peer review consultation*. undated-2014 online (no longer available): Swindon, UK.

84. Roebber, P.J. and D.M. Schultz, *Peer review, program officers and science funding.* PLoS ONE [Electronic Resource], 2011. **6**(4): p. e18680.

85. Ronai, Z., *Track record?* Pigment Cell & Melanoma Research, 2012. **25**(3): p. 291.

86. Russell, A.S., B.D. Thorn, and M. Grace, *Peer review: a simplified approach.* Journal of Rheumatology, 1983. **10**(3): p. 479-81.

87. Schroter, S., et al., *Effects of training on quality of peer review: randomised controlled trial.* BMJ, 2004. **328**(7441): p. 673.

88. Schroter, S., T. Groves, and L. Hojgaard, *Surveys of current status in biomedical science grant review: funding organisations' and grant reviewers' perspectives.* BMC Medicine, 2010. **8**: p. 62.

89. Sinkjær, T., *Fund ideas, not pedigree, to find fresh insight.* Nature, 2018. **555**(7695): p. 143.

90. Shepherd, J., et al., *Peer review of health research funding proposals: A systematic map and systematic review of innovations for effectiveness and efficiency.* PLOS ONE, 2018. **13**(5): p. e0196914.

91. Smith, M.A., N.J. Kaufman, and A.J. Dearlove, *External community review committee: a new strategy for engaging community stakeholders in research funding decisions.* Progress in Community Health Partnerships, 2013. **7**(3): p. 301-12.

92. Suls, J. and R. Martin, *The Air We Breathe: A Critical Look at Practices and Alternatives in the Peer-Review Process.* Perspectives on Psychological Science, 2009. **4**(1): p. 40-50.

93. Thornley, R., et al., *New decision tool to evaluate award selection process.* Journal of Research Administration, 2002. **33**(2/3): p. 49-58.

94. Whaley, A.L., *An objective rating form to evaluate grant proposals to the Hogg Foundation for Mental Health: a pilot study of implementation.* Evaluation Review, 2006. **30**(6): p. 803-16.

95. Wooding , S. and S. Guthrie, *Why We Need to Experiment with Grant Peer Review*. 2017, The RAND Blog.

96. Sattler DN, McKnight PE, Naney L, Mathis R. Grant peer review: improving inter-rater reliability with training. PloS One. 2015 Jun 15;10(6):e0130450.

1. **Characterisation of publications (n=95)**

| **Publication type** | | **Frequency**  **(% publications)** |
| --- | --- | --- |
|  | Original research | 51.6 |
|  | Review | 8.42 |
|  | Magazine | 8.42 |
|  | Report | 5.26 |
|  | Commentary | 8.42 |
|  | Editorial | 6.32 |
|  | Opinion letter | 5.26 |
|  | Analysis | 3.16 |
|  | Thesis | 1.05 |
|  | Blog | 2.11 |
| **Study design (research)** | |  |
|  | Observational study | 16.8 |
|  | Pilot | 14.7 |
|  | Modelling | 13.7 |
|  | Retrospective analysis | 12.6 |
|  | Review | 10.5 |
|  | Bibliometric analysis | 5.26 |
|  | Trial | 3.16 |
|  | Feasibility study | 1.05 |
|  | Prospective study | 1.05 |
| **Publication content (non-research)** | |  |
|  | Hypothetical narrative | 17.9 |
|  | Analysis of funder implementations | 4.21 |
|  | Analysis of funder recommendations | 4.21 |
|  | Analysis of funder reorganisation | 2.11 |
|  | Funder audit | 1.05 |
| **Research field(s)** | |  |
|  | Health/clinical | 48.4 |
|  | Field not specified | 42.1 |
|  | Biomedical | 36.8 |
|  | Natural and Physical sciences & Engineering | 15.8 |
|  | Social sciences & other | 8.42 |
|  | Science (not specified) | 6.32 |
| **Stakeholder involvement/relevance** | |  |
|  | Funder | 83.2 |
|  | Higher education institution | 50.5 |
|  | Research community & societies | 15.8 |
|  | Medical organisations & clinicians | 14.7 |
|  | Government bodies | 11.6 |
|  | National education ministries | 9.48 |
|  | External consultants & advisory groups | 5.26 |
|  | Journal editors | 1.05 |
| **Geographical location** | |  |
| USA | 53.7 |  |
| Europe | 22.1 |  |
| UK | 13.7 |  |
| Australia & New Zealand | 10.5 |  |
| Global (not specified) | 7.37 |  |
| Canada | 6.32 |  |
| Asia | 2.11 |  |

1. **Types of interventions by frequency of use**

| **Intervention type (n=45)** | | **Frequency of citation (% total publications)** |
| --- | --- | --- |
|  | Reviewer selection, rotation or proposal matching | 19 |
|  | Modifying review criteria | 17 |
|  | Modifying funding allocation | 16 |
|  | Reviewer or applicant training in PRDM | 16 |
|  | Modelling decision-making and Delphi | 15 |
|  | Reviewer or panel accountability | 14 |
|  | Bibliometrics | 14 |
|  | Shorter applications | 13 |
|  | Improving interrater reliability | 12 |
|  | Improving applicant feedback | 11 |
|  | Promoting interdisciplinary research | 9 |
|  | Introducing submission quotas | 8 |
|  | Virtual or remote panels and teleconferencing | 8 |
|  | Promoting innovation | 8 |
|  | Modifying scoring of proposals | 7 |
|  | Simplifying PRDM | 7 |
|  | Open access | 7 |
|  | Promoting early-career researchers | 6 |
|  | Patient and public involvement in PRDM | 6 |
|  | Reviewer blinding | 6 |
|  | Modifying panel composition or panel rotation | 6 |
|  | Promoting collaboration, research sponsoring and funder outreach | 6 |
|  | Continuous improvement of funding structures or processes | 5 |
|  | Random allocation of funding | 5 |
|  | Limiting resubmissions | 4 |
|  | Reviewer incentives | 4 |
|  | Golden ticket and autonomy in decision-making | 3 |
|  | Promoting clinical research | 3 |
|  | Increasing the application length | 2 |
|  | Secondary (applicant) allocation of funds | 2 |
|  | Promoting educational research | 2 |
|  | Promoting community engagement | 2 |
|  | Promoting career stability | 2 |
|  | Promoting new investigators | 2 |
|  | Promoting resubmissions | 1 |
|  | Proportionate reviewer quality (to the proposal quality) | 1 |
|  | Using multimedia in proposals | 1 |
|  | Standardising peer review | 1 |
|  | Maximising information use | 1 |
|  | Introducing classifications of criticisms in peer review | 1 |
|  | Automating funder processes | 1 |
|  | Limiting advertising for proposals | 1 |
|  | Transferring funding decisions to HEIs | 1 |
|  | Eliminating cronyism in decision-making | 1 |
|  | Sandpits | 1 |

1. **An update on recent funder interventions (n=36)**

| **Level of change** | **Intervention type** | **Funders implementing as of 2017-2020** |
| --- | --- | --- |
| **Impact-oriented changes to funding strategy** | Promoting innovation | UK Research Institutes^29^, National Science Foundation^10^, Economic and Social Research Council^7^, European Research Council^4^, Health Research Council of New Zealand^35^, National Institutes of Health^15^, National Institute for Health Research^21^, Lundbeck Foundation^18^, Japan Science and Technology Agency^25^, Q Community- The Health Foundation^12^ |
|  | Promoting early-career researchers | National Institutes of Health^15^, National Science Foundation^11^, European Research Council^4^, National Sciences and Engineering Research Council^27^, Health Research Council New Zealand^36^, UK Research Institutes^28^, National Institute for Health Research^21^, Japan Society for the Promotion of Science^26^ |
|  | Promoting interdisciplinary collaboration | UK Research Institutes^29^, National Health and Medical Research Council^13^, National Science Foundation^10^, Economic and Social Research Council^6^, Health Research Council of New Zealand^35^, Q Community- The Health Foundation^12^, Japan Science and Technology Agency^25^, Japan Society for the Promotion of Science^26^ |
|  | Promoting patient and public involvement in research | Wellcome Trust^32^, Department of Defense^5^, Association of Medical Research Councils^2^, Economic and Social Research Council^7^, National Sciences and Engineering Research Council^27^, National Institute for Health Research^19^, Q Community- The Health Foundation^12^ |
|  | Promoting open access, transparency and information sharing | UK Research Institutes^3^, Association of Medical Research Councils^2^, Health Research Council of New Zealand^36^, National Institute for Health Research^22^, Coalition S^24^ (International consortium of research funders) |
|  | Researcher training in research communication and peer review | National Institute for Health Research^20^, Natural Sciences and Engineering Research Council^27^, National Institutes of Health^14^ |
|  | Promoting gender equality | National Science Foundation^11^, European Research Council^4^ |
|  | Promoting multidisciplinary research  10 | National Science Foundation^10^, Health Research Council of New Zealand^34^ |
| **Organisation-level changes** | Promoting co-funding of research | National Science Foundation^10^, Economic and Social Research Council^7^, Health Research Council of New Zealand^34^, National Institute for Health Research^8^, Japan Science and Technology Agency^25^ |
|  | Introducing new award schemes | National Institutes of Health^15^, National Science Foundation^9^, Economic and Social Research Council^6^, Japan Science and Technology Agency^25^, Japan Society for the Promotion of Science^26^ |
|  | Promoting random allocation of funding | Health Research Council New Zealand^36^, Science for Technological Innovation^17^, Volkswagen Foundation^30^, Swiss National Science Foundation^1^ |
|  | Promoting use of virtual technology/panels | National Health and Medical Research Council^13^, National Science Foundation^9^, Health Research Council New Zealand^33^ |
|  | Continuous improvement of funding processes through research | Wellcome Trust^31^, National Institute for Health Research^23^ |
| **Incremental changes to PRDM** | Modifying the composition of review and decision panels | Department of Defense^5^, National Health and Medical Research Council^13^, Association of Medical Research Councils^2^, Economic and Social Research Council^7^, Natural Sciences and Engineering Research Council^27^ |
|  | Monitoring peer review | UK Research Institutes^7^, Department of Defense^5^, National Health and Medical Research Council^13^, Association of Medical Research Councils^2^ |
|  | Modifying review criteria | UK Research Institutes^7^, National Institutes of Health^16^, National Health and Medical Research Council^13^ |
|  | Modifying application format | UK Research Institutes^6^, National Institutes of Health^16^ |

References

[1-36]

1. Adam, D., Science funders gamble on grant lotteries. Nature, 2019. 575(7784): p. 574-575.

2. AMRC (Association of Medical Research Charities). *Balance, Accountability, Independence, Rotation, Impartiality: Raising the standards of research funding*. 2016  [cited 2020 January]; Available from: https://www.amrc.org.uk/Handlers/Download.ashx?IDMF=a7e3eead-67b0-49f6-9497-3ce472258d82.

3. University of St Andrews. *RCUK mandate compliance | University of St Andrews*. 2020  [cited 2020 January]; Available from: https://www.st-andrews.ac.uk/research/digitalresearch/openaccess/funderpolicies/rcuk/.

4. European Research Council. *ERC awards over €600 million to Europe's top researchers*. 2019 2019-12-05 [cited 2020 January]; Available from: https://erc.europa.eu/news/erc-awards-over-600-million-euro-europes-top-researchers.

5. Congressionally Directed Medical Research Programs - CDMRP. *CDMRP's Two-Tiered Review Process, About Us, Congressionally Directed Medical Research Programs*. 2020  [cited 2020 January]; Available from: https://cdmrp.army.mil/about/2tierRevProcess.

6. UKRI Economic Social Research Council. *Improving how you apply for funding*. 2020  [cited 2021 January]; Available from: https://www.ukri.org/apply-for-funding/before-you-apply/improving-how-you-apply-for-funding/.

7. UKRI Economic Social Research Council. *UKRI reducing unnecessary bureaucracy*. 2020  [cited 2021 January]; Available from: https://www.ukri.org/news/ukri-reducing-unnecessary-bureaucracy/.

8. National Institute for Health Research. Funding rapid research into COVID-19. 2020  [cited 2021 January]; Available from: https://www.nihr.ac.uk/covid-19/funding-urgent-research-into-covid-19-with-ukri.htm.

9. National Science Foundation. *New awards aim to improve online learning for today's workforce*. 2020  [cited 2020 January]; Available from: https://www.nsf.gov/news/special_reports/announcements/091919.jsp.

10. National Science Foundation. *NSF Convergence Accelerator awards bring together scientists, businesses, nonprofits to benefit workers*. 2020  [cited 2020 January]; Available from: https://www.nsf.gov/news/special_reports/announcements/091019.jsp.

11. National Science Foundation. *NSF awards $250 million to early career researchers*. 2020  [cited 2020 January]; Available from: https://www.nsf.gov/news/special_reports/announcements/080719.jsp.

12. Q Exchange - The Health Foundation. *Q Exchange | Q Community*. 2020  [cited 2020 January]; Available from: https://q.health.org.uk/get-involved/q-exchange/.

13. National Health Medical Research Council. *NHMRC’s peer review process is integral to supporting the best health and medical research in Australia*. 2020  [cited 2020 January]; Available from: https://www.nhmrc.gov.au/funding/peer-review.

14. National Institutes of Health. *2019 NIH Regional Seminar on Program Funding and Grants Administration*. 2019 2017-02-24 [cited 2020 January]; Available from: https://regionalseminars.od.nih.gov/phoenix2019/welcome/.

15. National Institutes of Health. *NIH Director's New Innovator Award*. 2020  [cited 2020 January]; Available from: https://www.ncbi.nlm.nih.gov/pubmed/.

16. National Institutes of Health. *Peer Review Policies and Practices | grants.nih.gov*. 2020  [cited 2020 January]; Available from: https://grants.nih.gov/policy/peer/index.htm.

17. Science for Technological Innovation. *Seed project funding 2020*. 2020  [cited 2021 January]; Available from: https://www.sftichallenge.govt.nz/for-researchers/apply-for-seed-project/.

18. Lundbeckfonden. *Lundbeck foundation awards grants worth dkk 57 million to bold projects*. 2020  [cited 2020 January]; Available from: https://www.lundbeckfonden.com/en/.

19. NIHR - National Institute for Health Research. *Involve patients | NIHR*. 2020  [cited 2020 January]; Available from: https://www.nihr.ac.uk/health-and-care-professionals/engagement-and-participation-in-research/involve-patients.htm.

20. NIHR - National Institute for Health Research. *Join the NIHR reviewer development scheme | NIHR*. 2020  [cited 2020 January]; Available from: https://www.nihr.ac.uk/health-and-care-professionals/career-development/reviewer-development-scheme.htm.

21. NIHR - National Institute for Health Research. *Learn about the Small Grants Scheme | NIHR*. 2020  [cited 2020 January]; Available from: https://www.nihr.ac.uk/health-and-care-professionals/engagement-and-participation-in-research/learn-about-the-small-grants-scheme.htm.

22. NIHR - National Institute for Health Research. *NIHR open access policy*. 2020  [cited 2020 January]; Available from: https://www.nihr.ac.uk/documents/nihr-open-access-policy/12251.

23. NIHR - National Institute for Health Research. *Peer Review: Can we do it better?* 2020  [cited 2020 January]; Available from: https://www.nihr.ac.uk/blog/peer-review-can-we-do-it-better/20096.

24. Plan S. *'Plan S' and 'cOAlition S' – Accelerating the transition to full and immediate Open Access to scientific publications*. 2020  [cited 2020 January]; Available from: https://www.coalition-s.org/.

25. Japan Science Technology Agency. *Japan Science and Technology Agency (JST)*. 2020  [cited 2020 January]; Available from: https://www.jst.go.jp/EN/index.html.

26. Japan Society for the Promotion of Science. *Programs of the Japan Society for the Promotion of Science*. 2020  [cited 2020 January]; Available from: https://www.jsps.go.jp/english/programs/index.html.

27. Natural Sciences and Engineering Research Council of Canada. *NSERC - Media Room - Latest News*. 2016 2016-06-28 [cited 2020 January]; Available from: https://www.nserc-crsng.gc.ca/Media-Media/News-Nouvelles_eng.asp.

28. RCUK (Research Councils UK). *RCUK statement on the responsible use of metrics in research assessment*. 2018; Available from: https://webarchive.nationalarchives.gov.uk/20180327101151/http://www.rcuk.ac.uk/media/news/180207/.

29. UKRI. *ESRC announces new funding for research methods training*. 2020  [cited 2021; Available from: https://webarchive.nationalarchives.gov.uk/20200930153832/https://esrc.ukri.org/news-events-and-publications/news/news-items/esrc-announces-new-funding-for-research-methods-training/.

30. VolkswagenStiftung. *Partially Randomized Procedure - Lottery and Peer Review | VolkswagenStiftung*. 2020  [cited 2020 January]; Available from: https://www.volkswagenstiftung.de/en/funding/our-funding-portfolio-at-a-glance/experiment/partially-randomized-procedure.

31. Wellcome. *Does the way Wellcome makes funding decisions support the right research?* 2019  [cited 2021; Available from: https://wellcome.org/news/does-way-wellcome-makes-funding-decisions-support-right-research.

32. Wellcome. *Redefining public engagement with science*. 2020  [cited 2020 January]; Available from: https://wellcome.org/news/redefining-public-engagement-science.

33. Australian Clinical Trials Alliance (ACTA). ACTA clinical trials webinar - Auckland. 2020  [cited 2021 January 2021]; Available from: https://www.hrc.govt.nz/news-and-events/events/acta-clinical-trials-webinar-auckland.

34. Health Research Council of New Zealand. *New funding for mid-career researchers now open*. 2020  [cited 2020 January]; Available from: https://www.hrc.govt.nz/news-and-events/new-funding-mid-career-researchers-now-open.

35. Health Research Council of New Zealand. *2020 e-ASIA Joint Research Program opens*. 2020  [cited 2020 January]; Available from: https://www.hrc.govt.nz/news-and-events/2020-e-asia-joint-research-program-opens.

36. Health Research Council of New Zealand. *Explorer grants*. 2021  [cited 2021 January]; Available from: https://gateway.hrc.govt.nz/funding/researcher-initiated-proposals/2021-explorer-grants.

1. **Search strategy in Medline (OVID)**

This strategy was adapted for use in other databases as necessary

Subject index terms

1 "peer review"/ or peer review, research/

2 (peer adj review*).tw.

3 1 or 2

4 (grant* adj2 (financ* or budget* or allocat*)).tw.

5 (research adj2 (fund* or grant* or proposal* or application* or applicant* or

submission* or budget* or financ*)).tw.

6 (program* adj grant*).tw.

7 (grant adj2 (application* or applicant* or submission*)).tw.

8 (grant adj2 proposal*).tw.

9 (grant adj2 award*).tw.

10 Financing, Organized/ or Financing, Government/

11 Research Support as Topic/ec

12 (fund* and decision*).tw.

13 (grant* and decision*).tw.

14 (protocol* adj5 (grant* or fund*)).tw.

15 Research Support as Topic/ec

16 Financial Management/

17 or/4‐16

18 3 and 17

19 ("peer review*" and grant*).ti.

20 ("peer review*" and fund*).ti.

21 18 or 19 or 20

22 ("peer review" and process* and research and grant*).tw.

23 ("peer review" and process* and research and fund*).tw.

24 21 or 22 or 23 (English language and human only)

1. **Initial programme theory statements under positive and negative perspectives for each hypothetical assumption**

| **If** | **then** |
| --- | --- |
| **If you have open peer review**  **Totally open (100% transparency)** | You have transparency  Openness  Honesty  Less rude/abrupt  Opportunities for development |
| **If you have open peer review** | You become more conscious/guarded  It can impact negatively on:   - career (senior researchers’ not appreciating honesty…) - Working relationships   It could raise bias from knowing who the applicants are |
| **If there is up to 3 external peer reviewers** | Time to locate reviewers decreases  Administrative burden in the organisation decreases  Number of invitations to review decreases  Number of opportunities for reviewers to mentor increase  Reviewing is more proportionate in terms of likelihood to try to improve an application |
| **If there is up to 3 external peer reviewers** | Risk of not having expertise to conduct a proper review  Increase burden (same as above) |
| **If external peer reviewers are part of the decision-making process** | There’s expertise in area of interest  Bias decreases  Opportunities to contribute to the field or society increase  It upholds scientific rigor  Provides a partial traceable decision process  Use of set process allows for more transparency and fairness – all applications treated to same criteria  Applicants would have the ability to rebut comments and improve application |
| **If external peer reviewers are part of the decision-making process** | Bias in specialist fields (e.g. rare diseases) increases  There is additional burden to applicants  Burden of staff identifying reviewer increases |
| **If we use proportionate (relevant sections) review** | Lessen burden and time for reviewer  Focus on expertise, conciseness, efficiency increase  (how) does it affect burden  Who, if benefit, does it benefit?  How does a review form look like? |
| **If we use proportionate (relevant sections) review** | Increase burden for funder  Potential increase scrutiny  Not impact on burden because it requires reviewing equal number of applications |
| **If proportionate review is used** | Time required to review applications decreases  Reduces decision-making processes  Increases turnaround time for some applications  Promotes sustainability of research community (reduce time for researchers to wait)  Makes research more timely  Increases pool of reviewers  Releases staff resources  More time to review less # applications [increase quality of review and decision confidence] |
| **If proportionate considering size of funding application (resources required)**  ***What is the right size?**  ***Relevance**  ***Study type** | Fund proposals of less scientific quality  Potential increase of duplication by not identifying research currently underway through reviewers  Increase researchers feeling that size is subjective not informative (fair) |
| **If (efficient) triage is in place**    **First gatepost**  **Where applications are assessed in terms of remit and competitiveness (proportionate cost to study)** | Applications that are not in remit would not go through, deceasing funder burden  Reduces time for applicants and funders – quick process that gives a y/n decision  Increases efficiency |
| **If triage is in place** | Staff training needs to develop appropriate expertise increase  Staff burden increases  Increases burden to funder  Increases time in the decision-making process  There is a hidden review process that could filter-out potentially good applications or let through bad applications |
| **If PPI is part of the decision –making**   - **Looking @full application** - **Being a member of FC** | Increase inclusivity  Ensures research has benefit and is of relevance to the public  Research is realistic – feasible for patients being recruited  There’s a view about value for money from the public  Reduce academic bias  Ensures there’s a balance between evidence generation and evidence users  Increases transparency & openness  The likelihood of identifying the most relevant research increases |
| **If PPI is part of the decision –making**   - **Looking at full application** - **As member of funding committee** | Risk of unbalanced weight in the role played by PPI in research increases  Increases likelihood of added participation of PPI active role in the research with cost implications |
